# β-catenin mutation reprograms ketone body metabolism to drive hepatocellular carcinoma metastasis and resistance to ketogenic therapy via transcriptional activation of OXCT1

**DOI:** 10.1038/s41419-026-08457-y

**Published:** 2026-03-09

**Authors:** Huan Li, Liyuan Qian, Yifan Ji, Yuanhao Geng, Yanjun Lu, Laizhu Zhang, Yanchao Xu, Weiwei Zong, Xiang Jiang, Xianwei Zhou, Jingyuan Wen, Donglin Liu, Ye Wang, Yunzheng Li, Binghua Li, Hucheng Ma, Decai Yu

**Affiliations:** 1 State Key Laboratory of Pharmaceutical Biotechnology, Division of Hepatobiliary and Transplantation Surgery, Department of General Surgery, Nanjing Drum Tower Hospital, Affiliated Hospital of Medical School, Nanjing University, Nanjing, China; 2 Department of Hepatobiliary and Pancreatic Surgery, The First People’s Hospital of Changzhou, The Third Affiliated Hospital of Soochow University, Changzhou, China; 3 Division of Hepatobiliary and Transplantation Surgery, Department of General Surgery, Nanjing Drum Tower Hospital Clinical College of Nanjing University of Chinese Medicine, Nanjing, China

**Keywords:** Cancer metabolism, Mechanisms of disease

## Abstract

The ketogenic diet is a controversial approach to cancer therapy. Over 30% of hepatocellular carcinoma (HCC) cases harbor β-catenin activating mutations, among which the S33Y mutation represents a classical hotspot conferring constitutive pathway activation. Our previous metabolic profiling predicted that β-catenin-mutated HCC may exhibit intrinsic resistance to ketogenic therapy. 3-oxoacid CoA-transferase 1 (OXCT1), the key enzyme for ketone body catabolism, is aberrantly expressed in β-catenin-mutated HCC. This study explores how β-catenin^S33Y^-mutated HCC activates OXCT1 to reprogram ketone body metabolism to drive HCC ketogenic therapy resistance and metastasis. Utilizing subcutaneous tumor models and patient-derived xenograft (PDX) models of HCC, we demonstrated that ketogenic treatment was effective in β-catenin-wild-type HCC, whereas β-catenin^S33Y^-mutated HCC exhibited ketogenic therapy resistance and increased metastasis. Mechanistically, mutated β-catenin^S33Y^ bound the transcription factor LEF1, which activated OXCT1 to promote ketolysis. An isotope metabolic flux experiment with C^13^-labeled β-hydroxybutyrate confirmed that β-catenin-activated OXCT1 converts ketone bodies into glutamate. Blocking OXCT1 in β-catenin^S33Y^-mutated HCC abolished resistance to ketogenic therapy and reduced tumor glutamate levels. Furthermore, OXCT1, activated by mutated β-catenin, enhanced HCC metastasis via the p-STAT3 and epithelial-mesenchymal transition pathways. Inhibition of OXCT1 attenuated its promoting effect on metastasis. Overall, in β-catenin^S33Y^-mutated HCC, OXCT1 activation leads to metabolic reprogramming of ketone bodies, resulting in resistance to ketogenic therapy and promoting metastasis. Targeting OXCT1 represents a promising strategy for treating β-catenin^S33Y^-mutated HCC.

## Introduction

Hepatocellular carcinoma (HCC) is the most common type of liver cancer, representing the third leading cause of cancer-related death [1]. Invasion, metastasis, and postoperative recurrence remain the primary causes of mortality in HCC patients [2], emphasizing the need for subtype-specific therapeutic strategies. Recent genomic studies revealed that aberrant activation of Wnt/β-catenin signaling is observed in 54% of HCC cases [3, 4], predominantly driven by activating mutations in β-catenin (encoded by CTNNB1). β-catenin activating mutations are detected in about 37% of HCCs and define a clinically distinct molecular subtype [4, 5]. Among these mutations, the S33Y mutation, located within the β-TrCP binding domain of CTNNB1 exon 3, represents a classical activating hotspot that impairs β-catenin phosphorylation and proteasomal degradation, resulting in constitutive pathway activation [6, 7]. Given the high frequency and oncogenic potential of this β-catenin^S33Y^-mutated subtype, elucidating its molecular vulnerabilities is essential for developing targeted therapeutic strategies in HCC.

β-catenin activation drives oncogenic transcriptional programs through TCF/LEF complexes [8]. and contributes to metabolic reprogramming of HCC cells, influencing fatty acid, glucose, and glutamine metabolism [9–11]. Our previous research has demonstrated that aberrant β-catenin activation reprograms ammonia metabolism and confers resistance to senescence in HCC. Furthermore, our metabolic profiling of HCC revealed that glycolytic subtypes (characterized by high glycolysis and low ketone body metabolism (KBM)) exhibit enhanced responsiveness to ketogenic diet therapy (KDT) compared to KB-metabolic subtypes (marked by high KBM and low glycolysis) [12]. Notably, the KB-metabolic subtypes were driven by β-catenin-activated mutations. Therefore, it is rational to hypothesize that β-catenin activation mutated HCC may display resistance in KDT.

KDT was initially used in the treatment of epilepsy [13]. Recently, a number of preclinical studies have confirmed that KDT is a promising therapeutic approach for tumors [14]. The ketogenic diet (KD) is a high-fat, low-carbohydrate diet with adequate protein and calories. KD produces ketone bodies, which are its active metabolites, and are also considered a necessary and adequate explanation for the anti-cancer effects [14, 15]. 3-oxoacid CoA-transferase 1 (OXCT1) is the sole catabolic enzyme for ketone bodies, and it is barely expressed in normal liver tissue, which means that the normal liver cannot utilize ketone bodies [16, 17]. it is markedly upregulated in HCC, suggesting a tumor-specific metabolic adaptation. Previous studies have shown that OXCT1 promotes HCC progression through protein succinylation, immune modulation, and metabolic compensation under nutrient stress [18–21]. However, the upstream mechanisms responsible for OXCT1 activation in β-catenin activation-mutated HCC remain unclear.

Here, we focused on β-catenin activation mutation HCC, particularly the classical S33Y mutant, to elucidate how β-catenin^S33Y^ activates OXCT1 to reprogram ketone body metabolism to drive HCC ketogenic therapy resistance and metastasis. We discovered that β-catenin^S33Y^ binding to LEF1 activates the transcription of OXCT1 in HCC. The activation of OXCT1 promotes ketone body catabolism, converting ketone bodies to glutamate, which leads to resistance to ketogenic therapy in β-catenin^S33Y^-mutated HCC. Additionally, β-catenin triggers OXCT1 to promote HCC metastasis via the p-STAT3/EMT pathways. Importantly, knockdown of OXCT1 significantly diminishes resistance to ketogenic therapy and metastatic potential in β-catenin^S33Y^-mutated HCC. Therefore, OXCT1 is an indispensable factor for metastasis and resistance to ketogenic therapy in β-catenin^S33Y^-mutated HCC.

## Materials and Methods

### Clinical samples

The paired tumor and adjacent non-tumor tissues were collected under the permission of patients with HCC undergoing partial hepatectomy in the Division of Hepatobiliary and Transplantation Surgery, Department of General Surgery, Nanjing Drum Tower Hospital. Informed consent was obtained from all patients.

### Ethics statement

This study was conducted in accordance with all relevant guidelines and regulations. The use of clinical samples was approved by the Research Ethics Committee of Drum Tower Hospital (Approval no. 2022-713). All animal experiments were approved by the Animal Ethics Committee of the Affiliated Drum Tower Hospital of Nanjing University (Protocol No. 2020AE01043).

### Cell line and culturing

The human HCC cell lines HCCLM3 (RRID: CVCL_6832) and Huh7 (RRID: CVCL_0336) were cultured in DMEM supplemented with 10% fetal bovine serum, 100 U/mL penicillin, and 100 μg/ml streptomycin (all from Thermofisher, USA). Cells were incubated at 37°C with 5% CO_2_.

### Infection protocol for recombinant lentivirus

Lentiviruses carrying overexpressing β-catenin^S33Y^ with flag labels were purchased from Shanghai Genechem Co., Ltd. Lentiviruses carrying overexpressing OXCT1 or shRNA targeting OXCT1 were purchased from OBiO Technology (Shanghai) Co., Ltd. Those lentiviruses infected HCCLM3 or Huh7 cells to get HCCLM3 cells stably expressing β-catenin (β-catenin) or non-targeting control (CON), Huh7 cells stably expressing OXCT1 shRNA (shOXCT1) or non-targeting control shRNA (shCON), HCCLM3 cells stable overexpressing β-catenin and Knockdown OXCT1 (β-catenin+shOXCT1).

### Cell transfection and dual-luciferase reporter assays

LEF1 and β-catenin^S33Y^ plasmid were purchased from Sino Biological (Beijing, China). The sequence of OXCT1 promoter (−2000— + 200) was cloned into the luciferase report plasmid pGL3-Basic and hence donated as PGL3-OXCT1-WT. Then, the sequence ACTTTGAACT (located at −1251— −1242) of the OXCT1 promoter was randomly mutated and named as pGL3-OXCT1-MUT. All cell transfections were performed by jetPRIME DNA & siRNA Transfection Reagent (polyplus-transfection, France) following the manual. After transfections for 48 h, luciferase activity was measured by Dual-Luciferase Reporter Gene Assay Kit (RG027, Beyotime, China) and normalized to the Renilla luciferase activity.

### Ketone body and glutamate measurements

The concentration of ketone bodies in the blood is determined using a blood β-ketone test strip (FreeStyle, Abbott). Intracellular β-Hydroxybutyrate content was measured by BHB Assay Kit (#700190, CaymanChemical) according to the manufacturer’s instructions. The glutamate content in cells and tissues is measured using a glutamate assay kit (#A074-1-2, Nanjing Jiancheng Bioengineering Institute, China).

### Co-inmunoprecipitation assay

LEF1 and β-catenin^S33Y^ plasmid were transfected into Huh7 cells. 48 h after transfection, Huh7 cells were lysed in IP lysis buffer (#P0013, Beyotime, China) containing protease inhibitor cocktail (#HY-K0010, MedChemExpress, China). The cell lysates were incubated with β-catenin antibody (#8480, Cell Signaling Technology, USA) at 4°C overnight. The antigen-antibody complex was coupled to the magnetic beads. The immunocomplex was subjected to immunoblotting using the LEF1 antibody (#2230, Cell Signaling Technology, USA), and corresponding secondary antibodies.

### Chromatin Immunoprecipitation Assay (ChIP)

The ChIP assay was performed through the ChIP Assay Kit (#P2078, Beyotime, China) following the manufacturer’s instructions. Briefly, Huh7 cells overexpressing LEF1 were cross-linked with 1% formaldehyde solution for 10 min at room temperature and incubated with 1.1 ml glycine solution (10X) for 5 min. DNA fragments ranging from 200 to 800 bp were obtained by ultrasonication. Then the lysate was immunoprecipitated with anti-LEF1 or IgG antibodies. Immunoprecipitated DNAs were analyzed by RT-qPCR.

### Metabolic flux analysis

HCCLM3 cells were treated with 20 μM SKL2001 or DMSO for 48 h followed by incubating with 5 mM [^13^C4] β-hydroxybutyrate (BHB) for 24 h, and subjected to UHPL-HRMS to measure metabolite changes of [^13^C4] BHB. Metabolite mass extraction, separation, and identification were commissioned to Shanghai Profleader Biotech Co., Ltd.

### Western Blot

Total proteins from cells and tissues were isolated using RIPA buffer (#P0013C, Beyotime) supplemented with protease inhibitor cocktail (#HY-K0010, MCE) and qualified by BCA detecting kit (#P0012, Beyotime, China). Equal amounts of proteins were separated by SDS-PAGE and transferred onto a PVD membrane. Primary and secondary antibodies were used to detect the targets on the membrane. Primary antibodies against the following proteins were used: β-actin (#20536-1-AP, Proteintech, China), OXCT1 (#12175-1-AP, Proteintech, China), β-catenin (#8480, Cell Signaling Technology, USA), PPARα (#66826-1-Ig, Proteintech, China), HMGCS2 (#ab137043, Abcam, USA), BDH1 (#15417-1-AP, Proteintech, China), LEF1 (#14972-1-AP, Proteintech, China), MMP2 (#10373-2-AP, Proteintech, China), Snai1 (13099-1-AP, Proteintech, China), Vimentin(#60330-1-Ig, Proteintech, China), E-cadherin (HY-P81271, MCE, China), STAT3 (#9139, Cell Signaling Technology, USA), p-STAT3 (#9145, Cell Signaling Technology, USA).

### RNA isolation and quantitative real-time PCR

Total RNA was extracted by TRIZOL (#R401, Vazyme, China) and reverse-transcribed using HiScript III RT SuperMix (#R323, Vazyme, China) according to the manufacturer’s protocol. Quantitative real-time PCR was performed using SYBR Green Master Mix (#Q711, Vazyme, China) on the Real-Time PCR system (Applied Biosystems QuantStudio 6 Real-Time PCR System, USA). The relative expression was normalized to β-actin by the 2^-ΔΔCt^ method.

### Wound healing and Transwell assay

Cells were grown to 70%-80% confluence. Then, the monolayer was scratched with a pipette tip and further cultured for 48 h. The width of the scratch was measured at 0 and 48 h to determine the migration rates of different experimental groups.

To measure the cell migration, 5.0 × 10^4^ cells suspended in serum-free medium were seeded into the upper compartment of Transwell inserts (Catalog #3422, Corning Biocoat). The lower compartment was supplemented with medium containing 20% fetal bovine serum (FBS). After a 24-hour incubation at 37 °C, the cells that had migrated to the lower surface of the membrane were fixed with paraformaldehyde, stained with crystal violet, and photographed to enumerate the number of migrated cells per experimental group. Cell invasion was similarly evaluated using Transwell inserts precoated with Matrigel (Catalog #354248, Corning Biocoat), with the procedure following the same steps as described above.

### Immunohistochemistry staining

Human and mouse HCC tissues were fixed in 4% paraformaldehyde, embedded in paraffin, sectioned, and subjected to antigen retrieval and blocking of non-specific staining. Sections were incubated overnight at 4 °C with primary antibodies against OXCT1 and Ki67. After three washes with TBS, sections were incubated with secondary antibodies and stained with hematoxylin.

### Animal Studies

Six-week-old male BALB/c nude and C57BL/6 mice (GemPharmatech, China) were used to establish HCC xenograft, intravenous metastasis, and hydrodynamic tail-vein injection models.

### Xenograft Experiment

HCCLM3-CON and HCCLM3-β-catenin cells (5×10^6^) were subcutaneously injected into the flanks of nude mice. Three days later, mice were randomly assigned to receive normal diets (ND) or ketogenic diets (KD) (#HF89.5, Dyets). Tumor size, body weight, blood glucose, and BHB levels were monitored every 3 days. Tumor volume was calculated as: tumor volume = (L×W²)/2. Blood was collected from tail veins for glucose and BHB measurements using a FreeStyle Optium Blood Glucose and Ketone Monitoring System (Abbott, UK). After 30 days, mice were euthanized and tumors were examined.

### Intravenous metastasis models

Liver cancer cells (2 × 10^6^) were injected into the tail vein of nude mice. After 45 days, mice were euthanized, and livers and lungs were harvested, fixed in formalin, sectioned, and stained with H&E for histological analysis.

### Hydrodynamic tail-vein injection models

The mouse orthotopic liver cancer induced by hydrodynamic tail vein injection (HTVi) of plasmids encoding NRas, β-catenin, and Sleeping Beauty transposase (SB), as described [22]. Plasmids (25 μg NRas, 25 μg β-catenin, and 2 μg SB) were dissolved in 2 mL physiological saline, filtered, and then injected into the lateral tail vein of 6 to 8-week-old C57BL/6 mice over 5 to 7 seconds.

### Patient-derived Xenograft (PDX) models

HCC PDX-β-catenin-WT models were established by implanting 2-3 mm³ tumor pieces into female NCG mice (GemPharmatech). Tumors were harvested when they reached 1500 mm³. Xenografts from F1 mice were implanted subcutaneously into 12 NCG mice. After 4 weeks, 6 mice with 200 mm³ tumors were randomly divided into two groups: one on a ketogenic diet and the other on a normal diet.

For the PDX-β-catenin-mut model, we commissioned Crown Bioscience to establish and test the effect of a ketogenic diet on β-catenin-mut tumors. Tumor tissues and sequencing data from patients with β-catenin-mutated HCC were provided by Crown Bioscience.

### Bioinformatic analysis

RNA sequencing and clinical data from multiple cancer types were retrieved from the NIH National Cancer Institute Genomic Data Commons (GDC) portal (https://portal.gdc.cancer.gov/). For hepatocellular carcinoma (HCC), patients from The Cancer Genome Atlas (TCGA) cohort were stratified into OXCT1-low (*n* = 185) and OXCT1-high (*n* = 185) groups based on the median expression level of OXCT1. Associations between OXCT1 expression and clinicopathological parameters were evaluated. Survival analysis was performed using the Kaplan–Meier method, and differences between groups were assessed by the log-rank test.

### Statistical analysis

All statistical analyses were performed using GraphPad Prism 9.0 and SPSS 24.0. For parametric tests, data were required to meet the assumptions of homogeneity of variance and normal distribution. Student’s *t*-test or Wilcoxon rank-sum test was used to compare the median values of two sets of continuous variables. The count data were analyzed with Pearson Chi-Square. Correlation between two continuous variables was measured by either Pearson’s r correlation or Spearman’s rank-order correlation. The repeated-measures ANOVA was used to compare the difference of the tumor volume in different groups. The log-rank test was used to determine the significance of differences between survival curves. The two-sided p-value less than 0.05 was regarded as indicative of statistical significance. The data are presented as mean ± standard deviation (SD).

## Results

### β-catenin^S33Y^ mutation promotes HCC progression and confers resistance to ketogenic therapy

**Fig. 1:**
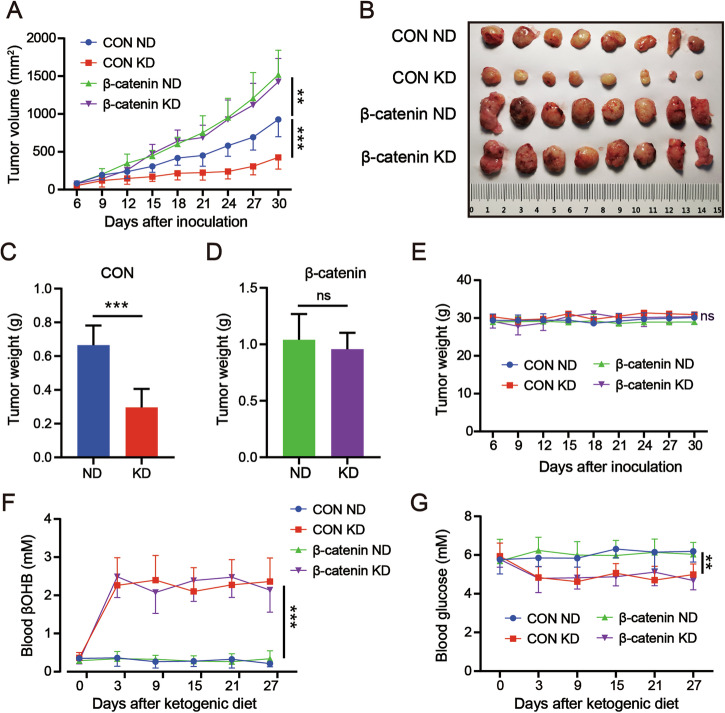
β-catenin^S33Y^ mutation promotes progression of HCC and ketogenic therapy resistance. HCCLM3 cells were infected with lentiviruses expressing β-catenin^S33Y^ or control viruses to establish stable HCCLM3-β-catenin and HCCLM3-CON cell lines. These cells were used to develop subcutaneous tumor models in mice (n = 8). The mice were then maintained on either ketogenic diets (KD) or normal diets (ND). **A** HCCLM3-CON and β-catenin^S33Y^ mutation cells were subcutaneously inoculated into the flank of nude mice. The tumor-bearing mice were random allocated to ND or KD groups. Six days after the injection, tumor volumes were monitored every three days. **B** Representative images of nude mice tumors stripped from each group. **C** Tumors weight of CON groups. **D** Tumors weight of β-catenin groups. **E** Body weight, **F** blood glucose, **G** Serum BHB levels of different groups of mice were monitored every three days and the curves were shown. Data are presented as mean ± SD. *, *p* < 0.01; **, *p* < 0.01; ***, *p* < 0.001; ns, not significant.

**Fig. 2:**
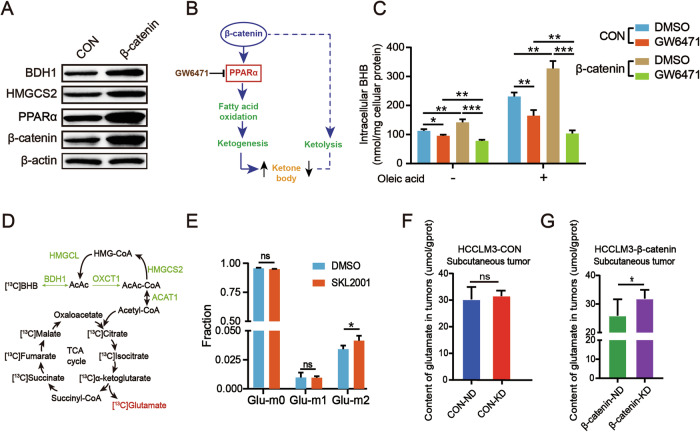
β-catenin^S33Y^ promotes ketolysis with glutamate formation in HCC cells. **A** Western blot analyzed the protein levels of β-catenin, PPARα, HMGCS2, and BDH1 in HCCLM3-CON and HCCLM3-β-catenin^S33Y^ mutation cells. **B** Model diagram of β-catenin regulating ketone body metabolism. **C** HCCLM3-CON and HCCLM3-β-catenin^S33Y^ cells were treated with PPARα-specific antagonist GW6471 (5 μM) or DMSO for 24 h followed by incubating with oleic acid or vehicle for 24 h. The BHB Assay Kit was used to measure the intracellular BHB of each group. **D** Schematic diagram of metabolic flux from ketone body metabolism to the TCA cycle, in which metabolites with [^13^C4] BHB-derived carbons measured were marked with ^13^C. **E** HCCLM3 cells treated with SKL2001 (20 μM) or DMSO for 48 h followed by incubating with 5 mM [^13^C4] BHB for 24 h. GC-MS analysis of ^13^C-labeled glutamate derived from [^13^C4] BHB in HCCLM3 cells. **F**, **G** Concentrations of glutamate in subcutaneous tumors from the experiment shown in Fig. 1. Data are presented as mean ± SD. *, *p* < 0.01; **, *p* < 0.01; ***, *p* < 0.001; ns, not significant.

HCCLM3 cells were infected with the β-catenin^S33Y^ lentivirus or a control virus to generate β-catenin^S33Y^ activating mutation HCCLM3-β-catenin (β-catenin) and HCCLM3-CON (CON) stable cell lines. WB analysis revealed significantly higher β-catenin expression in the β-catenin group compared to the CON group (Fig. 2A). HCCLM3-β-catenin and HCCLM3-CON cells were implanted subcutaneously in nude mice to form tumor models. The mice were maintained on either a normal diet (ND) or a ketogenic diet (KD). The results showed that β-catenin^S33Y^ mutation promoted the xenograft tumor growth whether on ND or KD (Fig. 1A, B). Ketogenic diet significantly inhibited tumor growth at the CON groups (CON ND vs CON KD) but had no tumor suppression in the β-catenin^S33Y^ mutation groups (β-catenin ND vs β-catenin KD), which suggests β-catenin^S33Y^ mutation led to ketogenic therapy resistance (Fig. 1A–D). Furthermore, our results showed KD had no effect on the body weight in nude mice (Fig. 1E) while it resulted in higher levels of serum ketone body (Fig. 1F) and lower levels of serum glucose (Fig. 1G) of CON and β-catenin groups. Taken together, these data demonstrated that β-catenin^S33Y^ mutation promoted HCC growth and induced resistance to ketogenic therapy in HCC.

### β-catenin^S33Y^ mutation promotes ketolysis with glutamate formation in HCC cells

How β-catenin^S33Y^ mutation lead to ketogenic therapy resistance in HCC? WB results indicated that in HCCLM3 cells, the activating mutation of β-catenin enhances the expression of ketone body metabolic enzymes BDH1, HMGCS2, and PPARα (Fig. 2A). Therefore, we investigated the effect of β-catenin^S33Y^ mutation on the metabolism of ketone bodies in the liver. We used β-hydroxybutyrate (BHB) detection kit to detect the concentration of ketone bodies. The result showed that oleic acid increased intracellular ketone levels (Fig. 2C). With or without oleic acid, BHB concentration was higher in β-catenin activation groups than in CON groups. As shown in the model diagram (Fig. 2B), β-catenin induced an enhanced PPARα-dependent FAO response to increase ketone bodies synthesis [23]. So utilized the PPARα-specific antagonist GW6471 to block the effect of β-catenin on ketone body synthesis. When both HCCLM3-CON and HCCLM3-β-catenin cells were treated with GW6471, the intracellular BHB content decreased. Interestingly, intracellular BHB levels of the β-catenin-GW6471 group were more significantly reduced than the CON-GW6471 group, which implied that β-catenin also participates in ketolysis in HCC cells. β-catenin regulates the catabolism of ketone bodies more obviously in the presence of oleic acid (Fig. 2C).

To further investigate the effect of β-catenin on ketone body metabolism, a ^13^C-BHB metabolic flux analysis was performed to trace the metabolites of ^13^C-BHB. HCCLM3 cells treated with Wnt/β-catenin agonists SKL2001 [24] or DMSO for 48 h, followed by incubation with 5 mM [^13^C4] BHB for an additional 24 h. Metabolites were then detected by GC-MS. The results show that the concentration of [^13^C4] glutamate was significantly higher in the β-catenin activation group than in the control group (Fig. 2E). Additionally, glutamate content was assessed in subcutaneous tumors from the experiment shown in Fig. 1. Notably, the β-catenin-KD group exhibited higher glutamate levels than the β-catenin-ND group, whereas no significant difference was observed between the CON-KD and CON-ND groups, which supported the metabolic flux findings (Fig. 2F, G). Collectively, these data demonstrated that β-catenin^S33Y^ mutation promotes the catabolism of ketone bodies and subsequent glutamate production in HCC.

### β-catenin binding LEF1 activates the transcription of OXCT1 expression in HCC cells

**Fig. 3:**
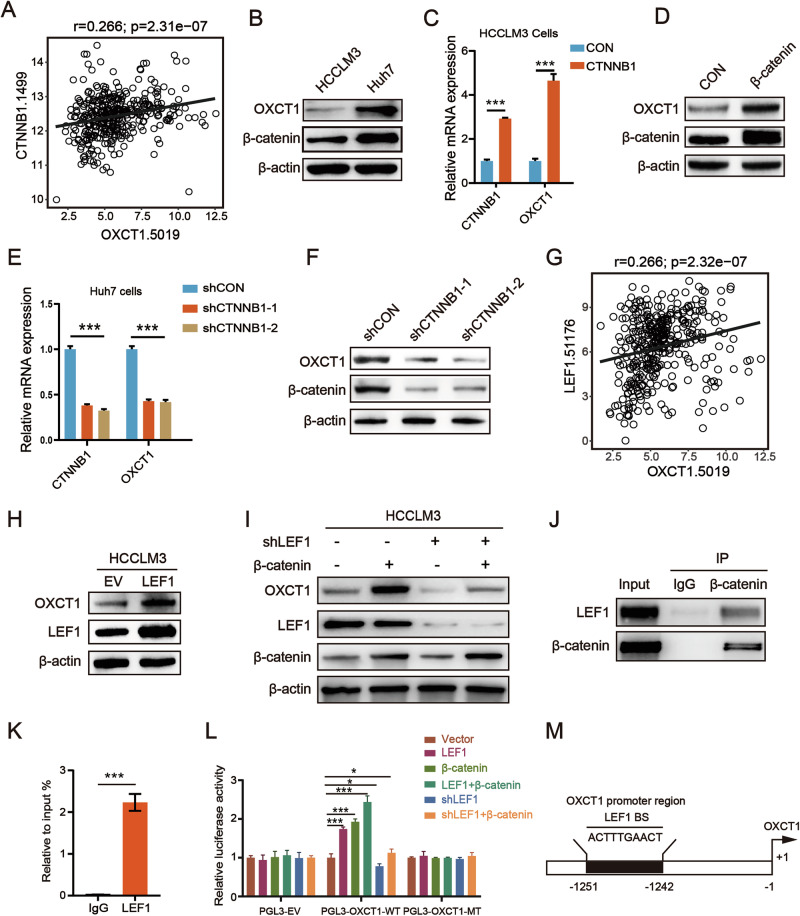
β-catenin^S33Y^ binding LEF1 activated the transcription of OXCT1 in HCC cells. **A** Correlation between OXCT1 and CTNNB1 (encoding β-catenin) RNA expression in HCC samples from the TCGA dataset. **B** Western blot analyzed the protein levels of β-catenin and OXCT1 in HCCLM3 and Huh7 cells. **C**, **D** The mRNA and protein levels of β-catenin and OXCT1 were analyzed by Western blot or RT-qPCR in HCCLM3-β-catenin^S33Y^ (β-catenin) or control (CON) cells. **E**, **F** The mRNA and protein levels of β-catenin and OXCT1 were analyzed by Western blot or RT-qPCR in Huh7 cells transfected with shRNA of CTNNB1 (shCTNNB) or no-targeting control (shCON). **G** Correlation between OXCT1 and LEF1 mRNA expression in HCC samples from the TCGA database. **H** Western blot analyzed protein levels of OXCT1 and LEF1 in HCCLM3 cells transfected with LEF1 plasmid or empty vector (EV). (**I**) Western blot analyzed protein levels of OXCT1 in HCCLM3 cells transfected with LEF1 shRNA (shLEF1) or β-catenin^S33Y^ vector. **J** Co-IP assay demonstrating the interaction between β-catenin and LEF1 in Huh7 cells. **K** ChIP assay was performed with LEF1 antibody. IgG was used as the negative control. Chip-PCR was conducted at the promoter regions of OXCT1 with Huh7 cells. **L** Dual-luciferase reporter assays in HEK293 cells. Cells were co-transfected with a wild-type (PGL3-OXCT1-WT) or mutant (PGL3-OXCT1-MT) OXCT1 promoter reporter, along with expression plasmids for β-catenin, LEF1, TCF4+β-catenin, or LEF1 shRNA, as indicated. Luciferase activity was measured 48 h post-transfection. **M** Schematic of the OXCT1 promoter region. The consensus LEF1-binding site (5′-ACTTTGAACT-3′) is indicated. Data are presented as mean ± SD. *, *p* < 0.05; **, *p* < 0.01; ***, *p* < 0.001, ns, not significant.

Given the exclusive role of OXCT1 in ketone body catabolism, we investigated whether β-catenin regulates ketolysis through OXCT1. Correlation analysis of the TCGA HCC cohort revealed a positive association between CTNNB1 and OXCT1 expression (Fig. 3A). OXCT1 was also significantly enriched in Wnt/β-catenin signaling gene sets (Fig. S1A). As β-catenin and OXCT1 were expressed at higher levels in Huh7 cells and at lower levels in HCCLM3 cells (Fig. 3B), we established HCCLM3 cells with an activating β-catenin^S33Y^ mutation and Huh7 cells with CTNNB1 knockdown. In β-catenin^S33Y^-mutant HCCLM3 cells, both OXCT1 protein and mRNA levels were elevated compared to control cells (Fig. 3C, D). Conversely, knockdown of CTNNB1 (encoding β-catenin) in Huh7 cells suppressed OXCT1 expression (Fig. 3E, F). Besides, treating HCCLM3 cells with SKL2001, an agonist of the Wnt/β-catenin signaling pathway, significantly increased OXCT1 protein levels (Fig. S1B-C). Treatment of Huh7 cells with XAV939, a tankyrase inhibitor of the Wnt/β-catenin signaling pathway [25], substantially decreased OXCT1 protein levels (Fig. S1D, E). The above results illustrated that β-catenin positively regulates OXCT1 expression in HCC cells.

We next explored the transcriptional mechanism. Bioinformatic screening using ALGGEN-PROMO and hTFtarget nominated LEF1 as a candidate transcriptional regulator of the OXCT1 promoter (Fig. 3G). Overexpression of LEF1 in HCCLM3 cells induced OXCT1 protein (Fig. 3H). Importantly, β-catenin-driven OXCT1 induction was abrogated upon LEF1 silencing, indicating that LEF1 is required for β-catenin-dependent OXCT1 upregulation (Fig. 3I). Co-immunoprecipitation confirmed a physical interaction between β-catenin and LEF1 in Huh7 cells (Fig. 3J). Chromatin immunoprecipitation demonstrated LEF1 occupancy at the OXCT1 promoter (Fig. 3K), and a putative LEF1 binding motif (ACTTTGAACT) was mapped to the −1251 to −1242 region of the promoter (Fig. 3M). Finally, dual-luciferase reporter assays showed that β-catenin and LEF1 cooperatively enhanced OXCT1 promoter activity, an effect that was abolished by LEF1 knockdown or mutation of the LEF1 binding site (Fig. 3L). Taken together, these results demonstrated that β-catenin activating mutation stimulated OXCT1 expression via the transcription factor LEF1.

### OXCT1 promotes the metastasis of HCC in vivo and in vitro

To investigate the role of OXCT1 activated by β-catenin in HCC, we first analyzed its clinical relevance. Analysis of the TCGA database revealed that OXCT1 is highly expressed in HCC (Fig. S2A). Immunohistochemical examination of samples from 144 HCC patients showed absent OXCT1 staining in normal liver tissues, with a positive OXCT1 expression observed in 25% (36/144) of tumor samples (Fig. S2B). Representative immunohistochemistry images demonstrate high OXCT1 expression in HCC (Fig. S2C). Western blot analysis confirmed significantly increased OXCT1 protein levels in tumor tissues compared to adjacent non-tumorous tissues (Fig. S2D). Additionally, analysis of the TCGA HCC dataset showed a negative correlation between OXCT1 expression in tumor tissues and patient prognosis (Fig. S2E and Supplementary Table 1). Collectively, these data suggest that OXCT1 activation may act as an oncogenic factor in HCC. Notably, Huang et al. demonstrated that OXCT1 activation promoted HCC cells' proliferation and growth under starvation [21, 26].

**Fig. 4:**
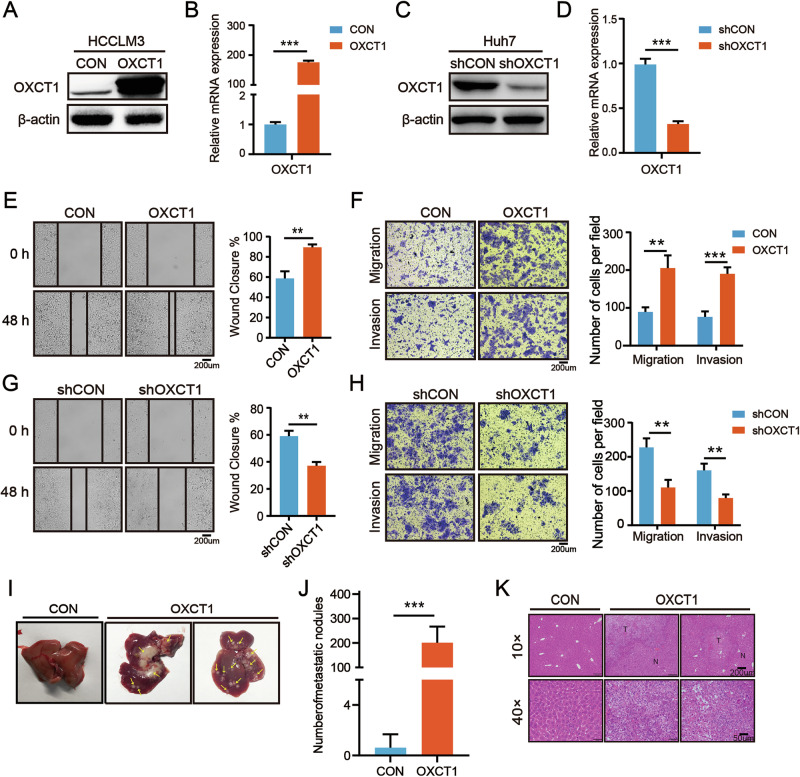
OXCT1 promotes HCC metastasis in vivo and in vitro. **A**, **B** The protein and mRNA levels of OXCT1 were analyzed by Western blot or RT-qPCR in HCCLM3 separately infected with OXCT1-overexpressing lentiviruses (OXCT1) and control lentiviruses (CON). **C**, **D** The protein and mRNA levels of OXCT1 were analyzed by Western blot or RT-qPCR in Huh7 separately infected with shOXCT1 lentiviruses (shOXCT1) and control lentiviruses (shCON). **E** Wound healing assay and **F** Transwell migration and invasion assays were performed in HCCLM3 separately infected with OXCT1-overexpressing lentiviruses (OXCT1) and control lentiviruses (CON). Quantified data are shown on the right. **G** Wound healing assay and **H** Transwell migration and invasion assays were performed in Huh7 separately infected with shOXCT1 lentiviruses (shOXCT1) and control lentiviruses (shCON). Quantified data are shown on the right. **I** In tail vein metastasis models, nude mice (*n* = 8 per group) were injected with 2 × 10⁶ HCCLM3-OXCT1 or HCCLM3-CON cells. Representative livers show intrahepatic metastases (arrows indicate lesions). **J** The counts of metastatic nodules were illustrated on the right panels. **K** Liver metastasis lesions were illustrated by HE staining. Data are presented as the mean ± SD. *, *p* < 0.05; **, *p* < 0.01; ***, *p* < 0.001, ns, not significant.

We next investigated whether activated OXCT1 has additional roles in HCC. Interestingly, we found that activated OXCT1 promoted metastasis in HCC. OXCT1 was overexpressed in HCCLM3 cells and knocked down in Huh7 cells. Successful overexpression (Fig. 4A, B) and knockdown (Fig. 4C, D) were confirmed. OXCT1 overexpression dramatically increased migration and invasive ability in HCCLM3-OXCT1 cells (Fig. 4E, F). In contrast, OXCT1 knockdown significantly suppressed the motility of Huh7 cells (Fig. 4G, H).

To assess the in vivo relevance, we performed tail vein metastasis assays. Mice injected with HCCLM3-OXCT1 cells developed significantly more and larger hepatic metastatic nodules than control mice (Fig. 4I, K; representative liver images and H&E staining), and quantification confirmed a significant increase in metastatic foci in the OXCT1 group (Fig. 4J). No significant difference in pulmonary metastasis was observed between groups (Fig. S3A-C). Collectively, these in vitro and in vivo data demonstrate that OXCT1 promotes HCC cell migration, invasion, and hepatic colonization, supporting a pro-metastatic role for OXCT1 in HCC.

### OXCT1 promotes HCC cells' migration and invasion via p-STAT3 and the EMT pathway

**Fig. 5:**
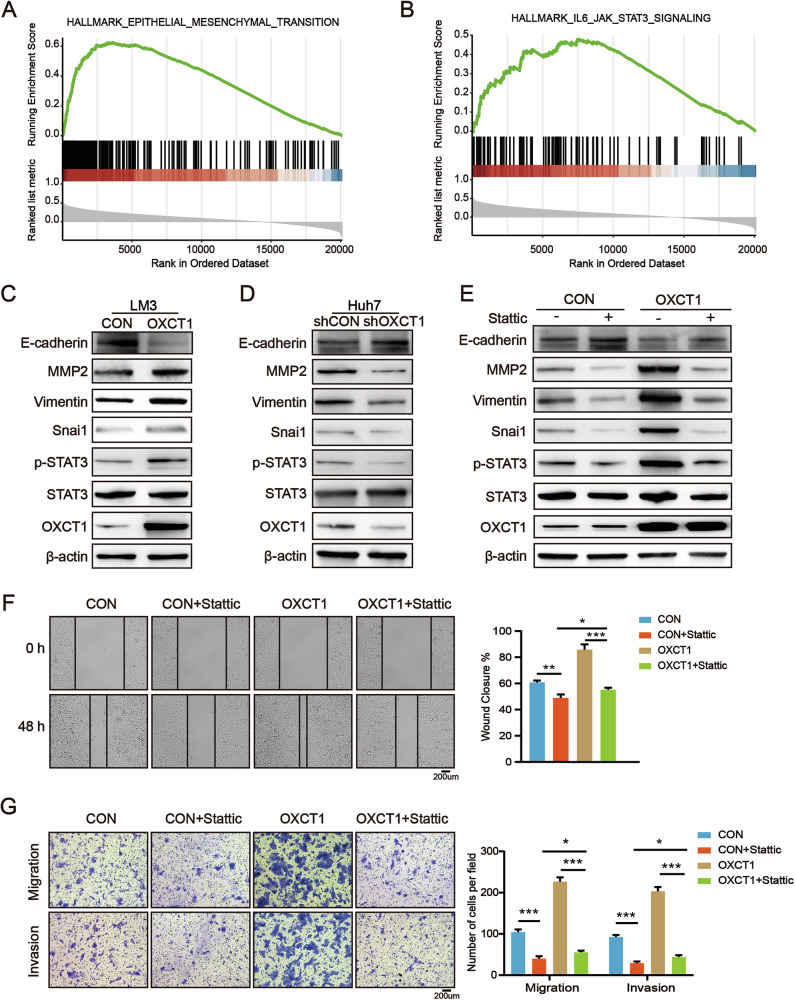
OXCT1 promotes HCC cells migration and invasion through p-STAT3/EMT axis. **A**, **B** Gene set enrichment analysis (GSEA) of the TCGA HCC cohort identified significant enrichment of EMT and IL-6/JAK/STAT3 signaling pathways in tumors with high OXCT1 expression. **C** Western blot analyzed protein levels of OXCT1, STAT3, p-STAT3, Snail, Vimentin, MMP2 and E-cadherin in HCCLM3-OXCT1 or CON cells and in (**D**) Huh7-shOXCT1 or shCON cells. **E** Western blot analyzed protein levels of OXCT1, STAT3, p-STAT3, Snail, Vimentin, MMP2 and E-Cadherin in HCCLM3 cells treated with vehicle (DMSO) or 10 μM Stattic (STAT3 Inhibitor) for 24 h. **F** Wound healing assay measured the migratory abilities of the HCCLM3 cells, respectively, treated with vehicle (DMSO) or 10 μM Stattic for 24 h. Quantified data are shown on the right. **G** Transwell migration and invasion assays of the HCCLM3 cells respectively, treated with vehicle (DMSO) or 10 μM Stattic for 24 h. Quantified data are shown on the right. Data are presented as the mean ± SD. *, *p* < 0.05; **, *p* < 0.01; ***, *p* < 0.001, ns, not significant.

To elucidate how OXCT1 promotes HCC cell migration and invasion, we performed gene set enrichment analysis (GSEA) on TCGA data, which revealed significant enrichment of epithelial-mesenchymal transition (EMT) and IL-6/JAK/STAT3 signaling in tumors with high OXCT1 expression (Fig. 5A, B). Given that STAT3 is a well-established upstream regulator of EMT across multiple cancers [26], we surveyed correlations between OXCT1 and canonical EMT markers (Snail1/2, Twist1/2, ZEB1/2, MMP2/9, and Vimentin) in the TCGA cohort, which confirmed strong positive associations (Fig. S4).

We next validated these associations in vitro. Western blotting showed that OXCT1 overexpression in HCCLM3 cells increased p-STAT3 and upregulated EMT effectors (Snail1, MMP2, and Vimentin) while reducing E-cadherin levels. Conversely, OXCT1 knockdown in Huh7 cells produced the opposite effects (Fig. 5C-D). When the upstream EMT regulator STAT3 was inhibited using Stattic [27], the expression of p-STAT3 and downstream EMT markers (Snail1, MMP2, and Vimentin) was markedly reduced, while E-cadherin expression was restored. Moreover, the migratory and invasive abilities of OXCT1-overexpressing cells were significantly impaired, as demonstrated by wound healing and transwell assays (Fig. 5E-G). Together, these data indicate that OXCT1 promotes HCC migration and invasion at least in part through activation of the STAT3 pathway and subsequent EMT program.

### OXCT1 is an indispensable mediator of β-catenin^S33Y^ mutation-driven HCC metastasis

**Fig. 6:**
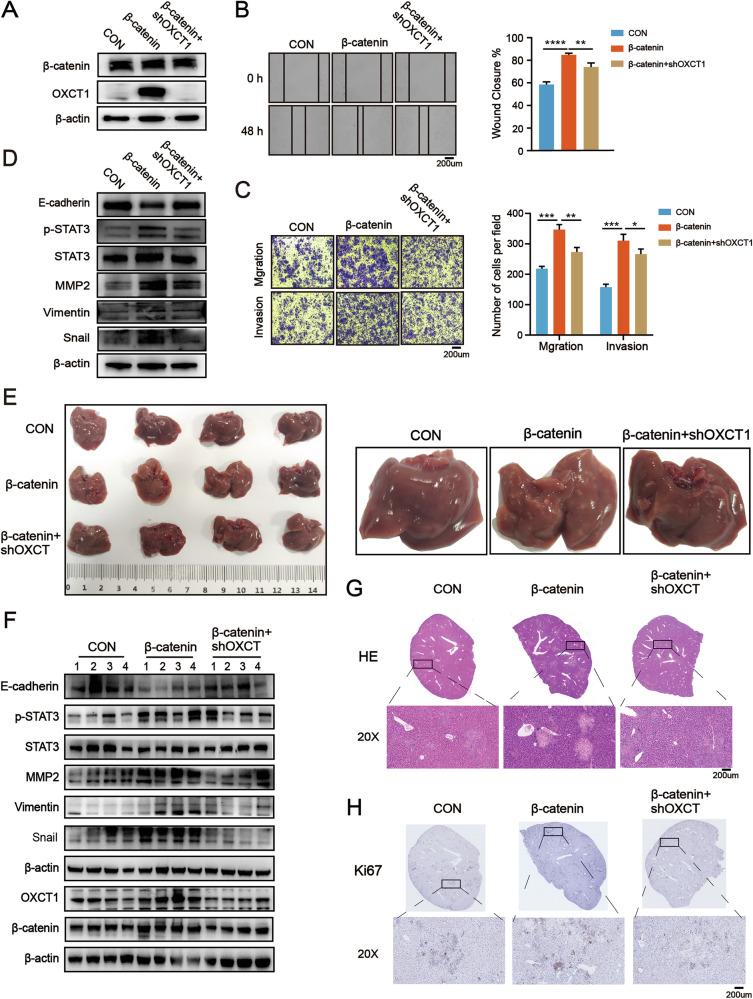
OXCT1 is an indispensable event for β-catenin^S33Y^ mutation to promote HCC metastasis. HCCLM3 cells stably overexpressing β-cateninS33Y (β-catenin) were further infected with shOXCT1 lentivirus to generate cells with concurrent OXCT1 knockdown (β-catenin+shOXCT1). Control (CON), β-catenin, and β-catenin+shOXCT1 cells were used. **A** Western blot analyzed protein levels of OXCT1 and β-catenin in the CON, β-catenin and β-catenin+shoxct1 groups. **B** Wound healing assay was performed to measure the migratory abilities of the indicated cell groups. **C** Transwell migration and invasion assays measured the migratory and invasive abilities of the indicated cell groups. **D** Western blot analyzed protein levels of STAT3, p-STAT3, MMP2, Snail, Vimentin and E-cadherin in the indicated cell groups. **E** In tail vein metastasis models, mice (*n* = 4 per group) were injected with 2 × 10⁶ cells from each group. Representative livers show metastatic burden (left panel: overview; right panel: magnified views of metastatic lesions). **F** Western blot analyzed protein levels of STAT3, p-STAT3, MMP2, Vimentin, Snail, E-cadherin, OXCT1, and β-catenin in liver metastatic tissues from each group. **G** Representative images of liver metastasis with HE staining of the CON, β-catenin, and β-catenin+shoxct1 groups. **H** Representative images of liver metastasis with Ki67 staining of the CON, β-catenin, and β-catenin+shoxct1 groups. Data are presented as the mean ± SD. *, *p* < 0.05; **, *p* < 0.01; ***, *p* < 0.001, ns, not significant.

To investigate whether β-catenin promotes HCC metastasis via OXCT1, we established HCCLM3 cells stably overexpressing β-catenin^S33Y^ (β-catenin) and those with concurrent OXCT1 knockdown (β-catenin + shOXCT1). Western blotting confirmed that OXCT1 expression was markedly induced by β-catenin and efficiently silenced in β-catenin + shOXCT1 cells (Fig. 6A). Functional assays showed that β-catenin^S33Y^ overexpression significantly enhanced cell migration and invasion, an effect largely abolished by concurrent OXCT1 knockdown (Fig. 6B, C). At the molecular level, β-catenin^S33Y^ overexpressing increased p-STAT3 and elevated EMT-associated proteins, including Snail, Vimentin, and MMP2, while reducing the epithelial marker E-cadherin. Silencing OXCT1 reversed these molecular changes, restoring E-cadherin expression and suppressing EMT activation (Fig. 6D).

In vivo, a tail vein metastasis model further demonstrated that β-catenin markedly promoted hepatic colonization, resulting in larger metastatic nodules and more extensive lesions, whereas OXCT1 knockdown significantly mitigated these effects (Fig. 6E, G). Western blot analysis of liver metastatic tissues confirmed that β-catenin increased p-STAT3 and EMT marker expression (Snail, Vimentin, and MMP2) and decreased E-cadherin, while OXCT1 depletion reversed these trends (Fig. 6F). Ki67 staining revealed higher proliferative indices in β-catenin tumors, which were reduced upon OXCT1 silencing (Fig. 6H). Pulmonary metastases were detected only in the β-catenin group but not in control or β-catenin + shOXCT1 mice (Fig. S5). Collectively, these in vitro and in vivo data demonstrate that OXCT1 is an indispensable event for β-catenin^S33Y^ activating mutation to promote HCC metastasis.

### β-catenin-mutant HCC confers resistance to ketogenic therapy via OXCT1

**Fig. 7:**
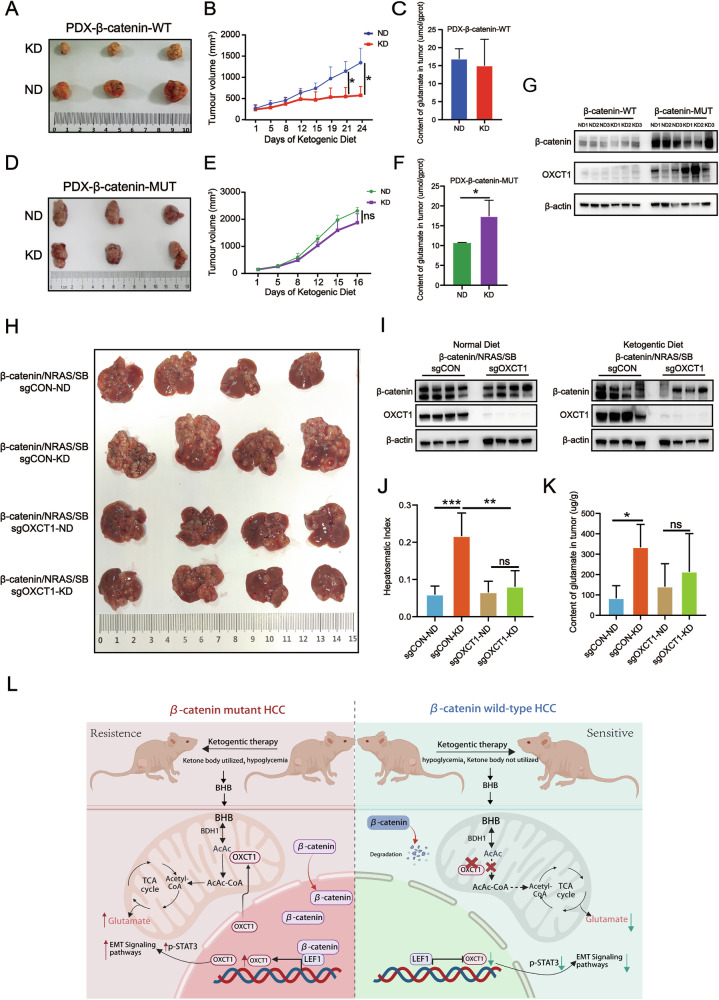
β-catenin-mutant HCC confers resistance to ketogenic therapy via OXCT1. Patient-derived xenograft (PDX) models were established from β-catenin wild-type (PDX-β-catenin-WT) and mutant (PDX-β-catenin-MUT) HCC tissues. Mice bearing these PDXs were fed a ketogenic diet (KD) or a normal diet (ND), and tumor volume was measured twice weekly (*n* = 3 per group). **A** Representative images of tumor tissues from the PDX-β-catenin-wild type (PDX-β-catenin-WT) model. **B** Tumor growth curves in the PDX-β-catenin-WT model. **C** Glutamate content in tumor tissues of the PDX-β-catenin-WT model. **D** Representative images of tumor tissues from the PDX-β-catenin-mutated (PDX-β-catenin-MUT) model. **E** Tumor growth curves in the PDX-β-catenin-MUT model. **F** Glutamate content in tumor tissues of the PDX-β-catenin-WT model. **G** Western blot analyzed protein levels of OXCT1and β-catenin in the PDX-β-catenin-WT and PDX-β-catenin-MUT model. An orthotopic liver cancer model was induced by hydrodynamic injection of an oncogenic plasmid cocktail (encoding β-catenin^S33Y^, NRAS, and Sleeping Beauty transposase). Mice were co-injected with CRISPR/sgRNA constructs targeting OXCT1 (sgOXCT1) or a non-targeting control (sgCON) and maintained on KD or ND (*n* = 4 per group). **H** Representative images of tumor tissues from the indicated groups. **I** Western blot analyzed protein levels of OXCT1 and β-catenin in the indicated groups. **J** Hepatosmatic Index (liver/body weight) of the indicated groups. **K** Glutamate content in tumor tissues of the indicated groups. **L** Graphic Abstract. Data are presented as the mean ± SD. *, *p* < 0.05; **, *p* < 0.01; ***, *p* < 0.001, ns, not significant.

In the first part, our results from subcutaneous tumor models demonstrate that β-catenin-mutant HCC is resistant to ketogenic therapy. To further validate this finding, we next evaluated the response of patient-derived xenograft (PDX) models to KD. We established PDX models from β-catenin wild-type (PDX-β-catenin-WT) and β-catenin-mutant (PDX-β-catenin-MUT) HCC specimens and administered either ND or KD. In PDX-β-catenin-WT mice, KD significantly suppressed tumor growth compared with ND (Fig. 7A-B; representative tumors and growth curves). By contrast, KD failed to inhibit the growth of PDX-β-catenin-MUT tumors (Fig. 7D, E), indicating that β-catenin-mutant HCC is KD resistant. Measurement of intratumoral glutamate revealed no significant change upon KD in WT PDXs (Fig. 7C), whereas it was significantly increased in KD-treated β-catenin-mutant PDXs (Fig. 7F), which is consistent with the glutamate changes observed in the subcutaneous tumors (Fig. 2F, G). Concordantly, OXCT1 protein expression was markedly higher in β-catenin-mutant tumors than in WT tumors (Fig. 7G), supporting that β-catenin activating mutation positively regulates OXCT1. These results suggest that resistance to ketogenic therapy in β-catenin-mutant HCC may be mediated by OXCT1 activation.

To directly test whether OXCT1 mediates KD resistance in β-catenin-mutant HCC in vivo, we used hydrodynamic tail-vein delivery of an oncogenic cassette (β-catenin/NRAS/SB) to induce orthotopic liver tumors and co-delivered either sgRNA targeting OXCT1 (sgOXCT1) or control sgRNA (sgCON). Mice were maintained on ND or KD, and tumor burden was assessed. In sgCON animals, KD exacerbated tumor progression, consistent with KD resistance in the β-catenin-activated context (Fig. 7H–J; representative livers and quantification). Importantly, OXCT1 knockout (sgOXCT1) substantially attenuated KD-associated tumor progression: KD failed to promote tumor growth in sgOXCT1 mice, and KD-treated sgOXCT1 tumors were significantly smaller than KD-treated sgCON tumors (Fig. 7H–J). Western blotting confirmed efficient OXCT1 depletion in sgOXCT1 tumors (Fig. 7I). Measurement of tumor glutamate levels showed that β-catenin activation increased ketone-to-glutamate conversion, whereas OXCT1 loss reduced this conversion (Fig. 7K). Therefore, OXCT1 is essential for the resistance of β-catenin^S33Y^-mutant HCC to ketogenic therapy.

## Discussion

As previously noted, 30-40% of HCC cases exhibit abnormal β-catenin activation due to mutations, making β-catenin-mutant HCC an important subtype. Our group previously discovered that β-catenin activating mutation in HCC promotes ammonia reprogramming by regulating GLUD1 and SLC4A11, thereby counteracting cellular senescence [10]. In this study, we report a novel finding: aberrantly activated β-catenin regulates ketone body catabolism via the sole ketone body-degrading enzyme OXCT1, which confers resistance to ketogenic therapy in β-catenin^S33Y^-mutant HCC. Furthermore, β-catenin-activated OXCT1 promotes HCC metastasis through the p-STAT and EMT pathways.

In recent years, ketogenic therapy has emerged as a prominent research focus, particularly for its applications in oncology [14, 28]. The ketogenic diet is being considered as a novel therapeutic approach for cancer treatment [14, 29]. However, its efficacy varies across different tumor types, creating some debate [14, 30]. Our team previously proposed a novel metabolic classification for tumors based on glycolytic and ketone body metabolic gene expression to predict clinical outcomes and therapeutic responses to ketogenic treatment [12]. This classification revealed that HCC patients with the KB-metabolic classification exhibit a significantly higher mutation rate in CTNNB1 (encoding mutations of β-catenin), suggesting a strong correlation between β-catenin mutations and ketogenic therapy resistance in HCC. To explore this, we established subcutaneous tumor models in mice using HCCLM3 overexpressing β-catenin^S33Y^ cells and HCCLM3-CON cells, and subjected them to either standard diets or ketogenic diets. Results showed that ketogenic therapy was effective in β-catenin^S33Y^ mutation HCC but ineffective in the control group. Additionally, we established HCC PDX models using β-catenin-wild-type and β-catenin-mutant HCC tissue from clinical patients, subjected these models to standard and ketogenic diets, and observed consistent results: ketogenic therapy was ineffective in β-catenin^S33Y^-mutant HCC. This experimental evidence provides the first direct support for the notion that β-catenin^S33Y^-mutant HCC is resistant to ketogenic therapy, validating our prior metabolic classification theory [12]. This finding highlights the importance of considering metabolic classification in ketogenic therapy for HCC.

Further investigation into ketone metabolism in β-catenin^S33Y^-mutant HCC revealed that β-catenin activation enhances ketone body catabolism, converting ketone bodies into glutamate. A study by Nadia Senni et al. proposed that in β-catenin^S33Y^-mutant HCC, fatty acid oxidation, mediated by PPARα, is upregulated, leading to an increase in acetyl-CoA and, subsequently, ketone body production [23]. This observation initially seemed contradictory to our findings. In fact, we blocked the pathway of β-catenin to promote ketone body synthesis by using the PPARα inhibitor GW647, ruling out the effect of synthesis. Our study thus provides the first evidence that β-catenin activation regulates ketone body catabolism in HCC, offering a more complete understanding of metabolic reprogramming of ketone bodies in β-catenin^S33Y^-mutant HCC.

We further elucidated the underlying mechanism. Specifically, we found that β-catenin transcriptionally upregulates OXCT1 expression in HCC cells. Activated β-catenin recruits TCF and LEF, forming a transcriptional complex that regulates downstream target gene transcription [31]. As a downstream target gene of β-catenin, the OXCT1 promoter region was predicted to contain a binding site for the transcription factor LEF1. Using Co-IP, ChIP, and dual-luciferase reporter assays, we confirmed that OXCT1 is a direct transcriptional target of β-catenin, whose expression is driven by the β-catenin/LEF1 complex.

Research on OXCT1 in tumors remains limited, with most studies focusing on its canonical function in ketone body catabolism. Recently, OXCT1 overexpression was found to promote the growth and metastasis of breast cancer cells, although the underlying mechanisms remain unclear [32]. In HCC, Zhang et al. reported that serum starvation (SS) activates OXCT1 expression, which, in turn, promotes HCC cell proliferation [21]. Our study identifies a novel function of OXCT1 in facilitating HCC metastasis. Specifically, in β-catenin^S33Y^-mutated HCC, OXCT1 is activated and modulates the p-STAT3 and EMT pathways, thereby promoting cancer cell metastasis. Furthermore, OXCT1 is an indispensable mediator of β-catenin-activated HCC metastasis. Consistently, β-catenin-mutated HCC demonstrates resistance to ketogenic therapy via OXCT1 activation, highlighting its pivotal role in both tumor progression and therapeutic resistance.

In summary, in β-catenin^S33Y^-mutated HCC, aberrantly activated β-catenin binds to the transcription factor LEF1, thereby activating the transcription of OXCT1. This leads to enhanced ketone body catabolism, which confers resistance to ketogenic therapy. Concurrently, OXCT1 activation drives HCC metastasis through the p-STAT3/EMT pathways. Therefore, OXCT1 is a central node mediating both therapeutic resistance and metastasis in β-catenin^S33Y^-mutant HCC. Targeting OXCT1 will be a new strategy for the treatment of β-catenin^S33Y^-mutant HCC.

## Supplementary information

Supplementary Figures

Supplementary Table 1: Analysis of the Correlation between OXCT1 Expression and Clinicopathological Data

Original Western blots

## Data Availability

The data generated in this study are available within the article and its supplementary data files. Additional data are available from the corresponding author upon reasonable request.

## References

[1] Llovet JM, Kelley RK, Villanueva A, Singal AG, Pikarsky E, Roayaie S, et al. Hepatocellular carcinoma. Nat Rev Dis Prim. 2021;7:6. 33479224 10.1038/s41572-020-00240-3 PMC13537433

[2] Kulik L, El-Serag HB. Epidemiology and management of hepatocellular carcinoma. Gastroenterology. 2019;156:477–91.e1. 30367835 10.1053/j.gastro.2018.08.065 PMC6340716

[3] Li B, Cao Y, Meng G, Qian L, Xu T, Yan C, et al. Targeting glutaminase 1 attenuates stemness properties in hepatocellular carcinoma by increasing reactive oxygen species and suppressing Wnt/beta-catenin pathway. EBioMedicine. 2019;39:239–54. 30555042 10.1016/j.ebiom.2018.11.063 PMC6355660

[4] Russell JO, Monga SP. Wnt/beta-Catenin signaling in liver development, homeostasis, and pathobiology. Annu Rev Pathol. 2018;13:351–78. 29125798 10.1146/annurev-pathol-020117-044010 PMC5927358

[5] Zhang Y, Wang X. Targeting the Wnt/beta-catenin signaling pathway in cancer. J Hematol Oncol. 2020;13:165. 33276800 10.1186/s13045-020-00990-3 PMC7716495

[6] Wang Z, Li Z, Ji H. Direct targeting of beta-catenin in the Wnt signaling pathway: Current progress and perspectives. Med Res Rev. 2021;41:2109–29. 33475177 10.1002/med.21787 PMC8217106

[7] Rebouissou S, Franconi A, Calderaro J, Letouze E, Imbeaud S, Pilati C, et al. Genotype-phenotype correlation of CTNNB1 mutations reveals different ss-catenin activity associated with liver tumor progression. Hepatology. 2016;64:2047–61. 27177928 10.1002/hep.28638

[8] He S, Tang S. WNT/beta-catenin signaling in the development of liver cancers. Biomed Pharmacother. 2020;132:110851. 33080466 10.1016/j.biopha.2020.110851

[9] Montagner A, Le Cam L, Guillou H. beta-catenin oncogenic activation rewires fatty acid catabolism to fuel hepatocellular carcinoma. Gut. 2019;68:183–5. 30077995 10.1136/gutjnl-2018-316557

[10] Wang Y, Cheng C, Lu Y, Lian Z, Liu Q, Xu Y, et al. beta-Catenin activation reprograms ammonia metabolism to promote senescence resistance in hepatocellular carcinoma. Cancer Res. 2024;84:1643–58. 38417136 10.1158/0008-5472.CAN-23-0673

[11] Monga SP. beta-Catenin signaling and roles in liver homeostasis, injury, and Tumorigenesis. Gastroenterology. 2015;148:1294–310. 25747274 10.1053/j.gastro.2015.02.056 PMC4494085

[12] Qian L, Li Y, Cao Y, Meng G, Peng J, Li H, et al. Pan-cancer analysis of glycolytic and ketone bodies metabolic genes: implications for response to ketogenic dietary therapy. Front Oncol. 2021;11:689068. 34692477 10.3389/fonc.2021.689068 PMC8529115

[13] Borowicz-Reutt K, Krawczyk M, Czernia J. Ketogenic diet in the treatment of epilepsy. Nutrients. 2024;16. 10.3390/nu16091258 PMC11085120 38732505

[14] Weber DD, Aminzadeh-Gohari S, Tulipan J, Catalano L, Feichtinger RG, Kofler B. Ketogenic diet in the treatment of cancer - Where do we stand?. Mol Metab. 2020;33:102–21. 31399389 10.1016/j.molmet.2019.06.026 PMC7056920

[15] Puchalska P, Crawford PA. Metabolic and signaling roles of ketone bodies in health and disease. Annu Rev Nutr. 2021;41:49–77. 34633859 10.1146/annurev-nutr-111120-111518 PMC8922216

[16] Zhang J, Jia PP, Liu QL, Cong MH, Gao Y, Shi HP, et al. Low ketolytic enzyme levels in tumors predict ketogenic diet responses in cancer cell lines in vitro and in vivo. J Lipid Res. 2018;59:625–34. 29414764 10.1194/jlr.M082040 PMC5880499

[17] Zhang S, Xie C. The role of OXCT1 in the pathogenesis of cancer as a rate-limiting enzyme of ketone body metabolism. Life Sci. 2017;183:110–5. 28684065 10.1016/j.lfs.2017.07.003

[18] Ma W, Sun Y, Yan R, Zhang P, Shen S, Lu H, et al. OXCT1 functions as a succinyltransferase, contributing to hepatocellular carcinoma via succinylating LACTB. Mol Cell. 2024;84:538–51 e7. 38176415 10.1016/j.molcel.2023.11.042

[19] Guo D, Yu Q, Tong Y, Qian X, Meng Y, Ye F, et al. OXCT1 succinylation and activation by SUCLA2 promotes ketolysis and liver tumor growth. Mol Cell. 2025;85:843–56 e6. 39862868 10.1016/j.molcel.2024.12.025

[20] Zhu CX, Yan K, Chen L, Huang RR, Bian ZH, Wei HR, et al. Targeting OXCT1-mediated ketone metabolism reprograms macrophages to promote antitumor immunity via CD8(+) T cells in hepatocellular carcinoma. J Hepatol. 2024;81:690–703. 38759889 10.1016/j.jhep.2024.05.007

[21] Huang D, Li T, Wang L, Zhang L, Yan R, Li K, et al. Hepatocellular carcinoma redirects to ketolysis for progression under nutrition deprivation stress. Cell Res. 2016;26:1112–30. 27644987 10.1038/cr.2016.109 PMC5113304

[22] Lee SA, Ho C, Roy R, Kosinski C, Patil MA, Tward AD, et al. Integration of genomic analysis and in vivo transfection to identify sprouty 2 as a candidate tumor suppressor in liver cancer. Hepatology. 2008;47:1200–10. 18214995 10.1002/hep.22169

[23] Senni N, Savall M, Cabrerizo Granados D, Alves-Guerra MC, Sartor C, Lagoutte I, et al. beta-catenin-activated hepatocellular carcinomas are addicted to fatty acids. Gut. 2019;68:322–34. 29650531 10.1136/gutjnl-2017-315448

[24] Gwak J, Hwang SG, Park HS, Choi SR, Park SH, Kim H, et al. Small molecule-based disruption of the Axin/beta-catenin protein complex regulates mesenchymal stem cell differentiation. Cell Res. 2012;22:237–47. 21826110 10.1038/cr.2011.127 PMC3351914

[25] Huang SM, Mishina YM, Liu S, Cheung A, Stegmeier F, Michaud GA, et al. Tankyrase inhibition stabilizes axin and antagonizes Wnt signalling. Nature. 2009;461:614–20. 19759537 10.1038/nature08356

[26] Jin W Role of JAK/STAT3 signaling in the regulation of metastasis, the transition of cancer stem cells, and chemoresistance of cancer by epithelial-mesenchymal transition. Cells. 2020;9. 10.3390/cells9010217 PMC7017057 31952344

[27] Schust J, Sperl B, Hollis A, Mayer TU, Berg T. Stattic: a small-molecule inhibitor of STAT3 activation and dimerization. Chem Biol. 2006;13:1235–42. 17114005 10.1016/j.chembiol.2006.09.018

[28] Weber DD, Aminazdeh-Gohari S, Kofler B. Ketogenic diet in cancer therapy. Aging. 2018;10:164–5. 29443693 10.18632/aging.101382 PMC5842847

[29] Talib WH, Mahmod AI, Kamal A, Rashid HM, Alashqar AMD, Khater S, et al. Ketogenic diet in cancer prevention and therapy: molecular targets and therapeutic opportunities. Curr Issues Mol Biol. 2021;43:558–89. 34287243 10.3390/cimb43020042 PMC8928964

[30] Mundi MS, Mohamed Elfadil O, Patel I, Patel J, Hurt RT. Ketogenic diet and cancer: Fad or fabulous?. JPEN J Parenter Enter Nutr. 2021;45:26–32. 10.1002/jpen.2226 34897736

[31] Hovanes K, Li TW, Munguia JE, Truong T, Milovanovic T, Lawrence Marsh J, et al. Beta-catenin-sensitive isoforms of lymphoid enhancer factor-1 are selectively expressed in colon cancer. Nat Genet. 2001;28:53–7. 11326276 10.1038/ng0501-53

[32] Martinez-Outschoorn UE, Lin Z, Whitaker-Menezes D, Howell A, Sotgia F, Lisanti MP. Ketone body utilization drives tumor growth and metastasis. Cell Cycle. 2012;11:3964–71. 23082722 10.4161/cc.22137 PMC3507492

