# Supplementary material for: β-catenin mutation reprograms ketone body metabolism to drive hepatocellular carcinoma metastasis and resistance to ketogenic therapy via transcriptional activation of OXCT1
Source: Cell Death Dis. 2026 Mar 9;17(1):301. doi: 10.1038/s41419-026-08457-y (PMC13039260; doi:10.1038/s41419-026-08457-y)
Supplement: Supplementary file 1 — Supplementary Figures [file 41419_2026_8457_MOESM1_ESM.docx]

**Supplementary Figure 1**


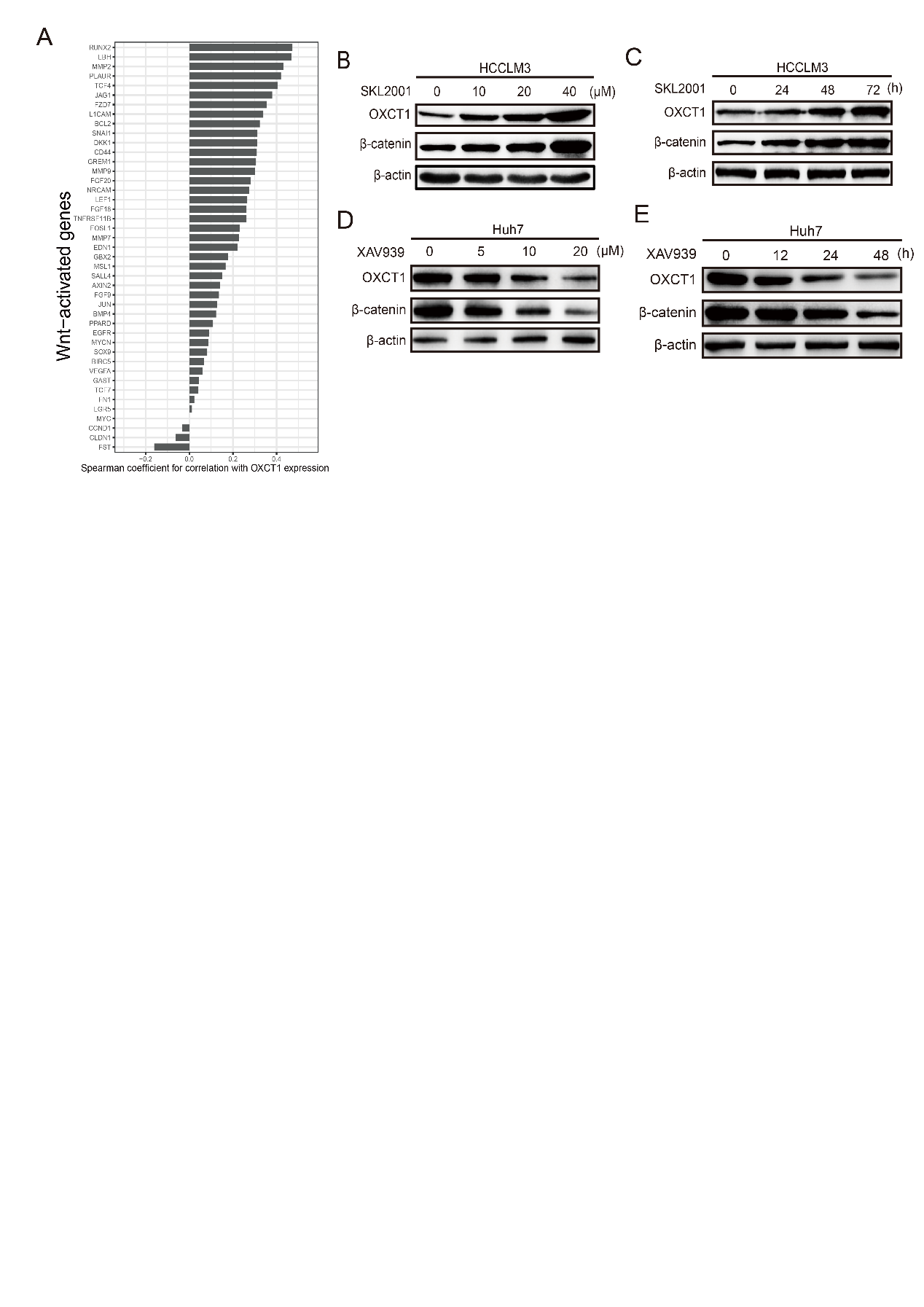


**Supplementary Figure 1. β-catenin regulated OXCT1 expression in HCC cells.** (A) Spearman correlation analysis between OXCT1 mRNA and established Wnt target genes (source: Wnt signaling pathway database, https://web.stanford.edu/group/nusselab/cgi-bin/wnt) in the TCGA LIHC cohort. Wnt-upregulated transcripts showed an almost exclusively positive correlation with OXCT1 expression. (B) Protein levels of β-catenin and OXCT1 in HCCLM3 cells treated with the indicated concentrations of the Wnt/β-catenin agonist SKL2001 for 48 h. (C) Western blot analyzed the protein levels of β-catenin and OXCT1 in HCCLM3 cells treated with the Wnt/β-catenin signaling pathway agonist SKL2001(10μM) for the indicated time. (D) Western blot analyzed the protein levels of β-catenin and OXCT1 in Huh7 cells treated with the indicated concentration of Wnt/β-catenin signaling pathway inhibitor XAV939 for 24 h. (E) Western blot analyzed the protein levels of β-catenin and OXCT1 in Huh7 cells treated with the 10μM XAV939 for the indicated time.

**Supplementary Figure 2**


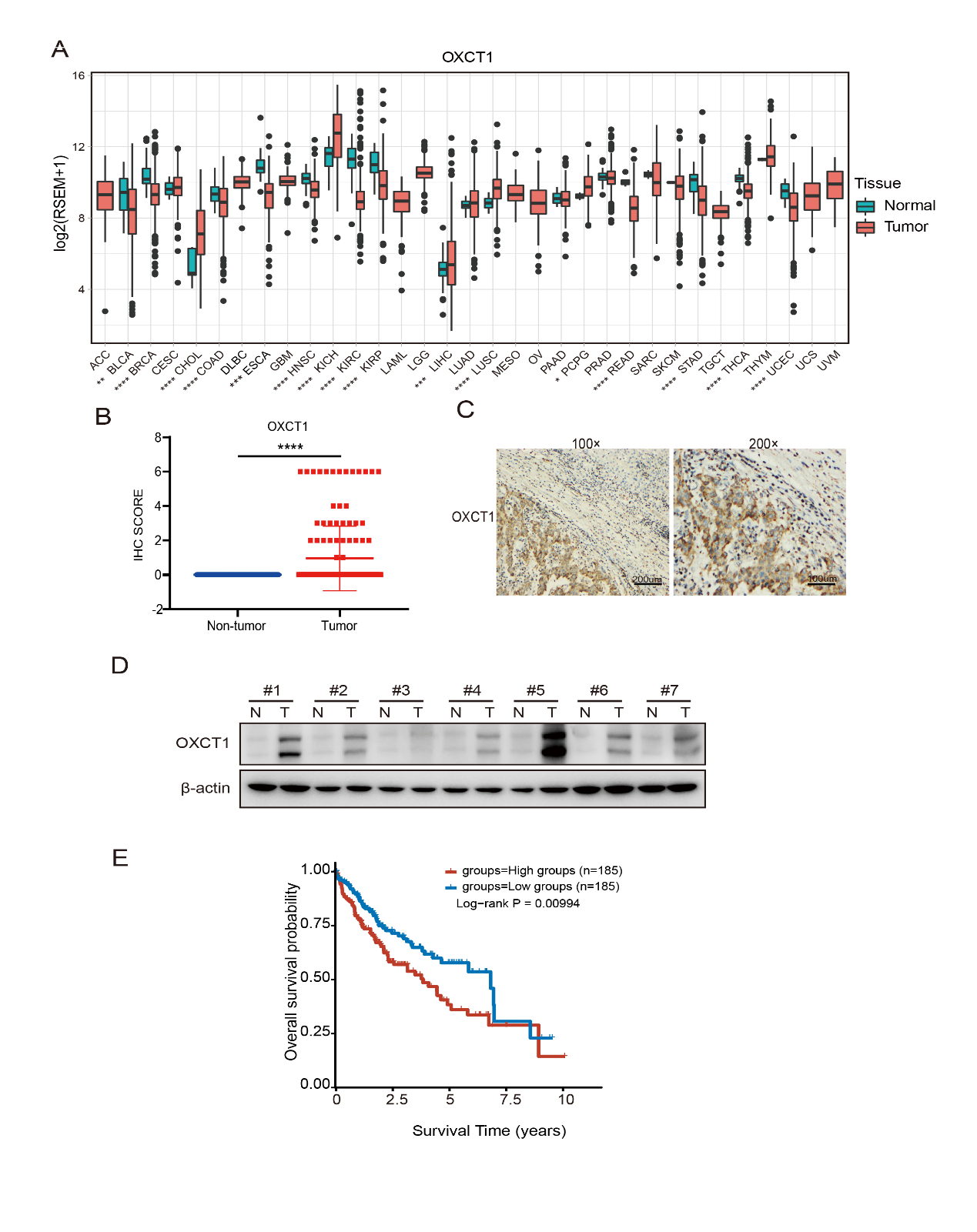


**Supplementary Figure 2. OXCT1 is upregulated and associated with poor prognosis in HCC.** (A) Pan-cancer analysis of OXCT1 mRNA expression across tumor types in the TCGA database. (B) OXCT1 expression in HCC tissues was assessed by immunohistochemical staining. (C) Representative IHC images showing OXCT1 expression in HCC and matched adjacent non-tumor tissues. (D) Western blot analysis of OXCT1 expression in HCC and matched non-tumor tissues (N: normal liver tissue; C: liver cancer tissue). (E) Kaplan-Meier survival analysis of HCC patients stratified by high or low OXCT1 mRNA expression (based on consensus clustering of the TCGA-LIHC cohort). Data are presented as the mean ± SD. *, p <0.05; **, p <0.01; ***, p <0.001; ****, p <0.0001.

**Supplementary Figure 3**


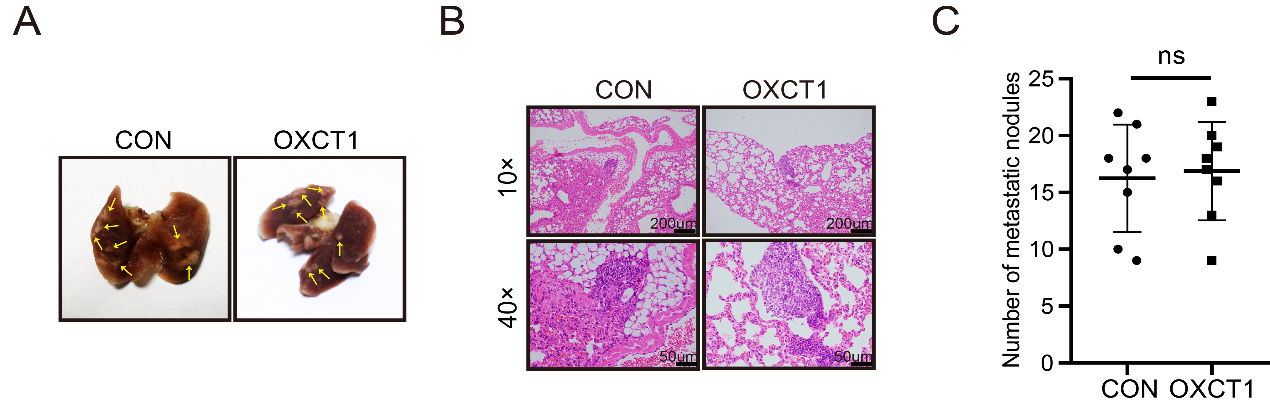


**Supplementary Figure 3. OXCT1-activated HCC cells showed no difference in lung metastasis.** For metastasis models via intravenous tail vein injections, 2×10^6^ HCCLM3-OXCT1 or CON cells were injected into the tail vein of nude mice (200 μl per mouse, n=8 per group) (A) Representative lung tissues from mice in the indicated groups. Arrows indicate metastatic lesions. (B) H&E staining of lung metastatic lesions. (C) Quantification of metastatic nodules in the lung. Data are presented as the mean ± SD. ns. not significant.

**Supplementary Figure 4**


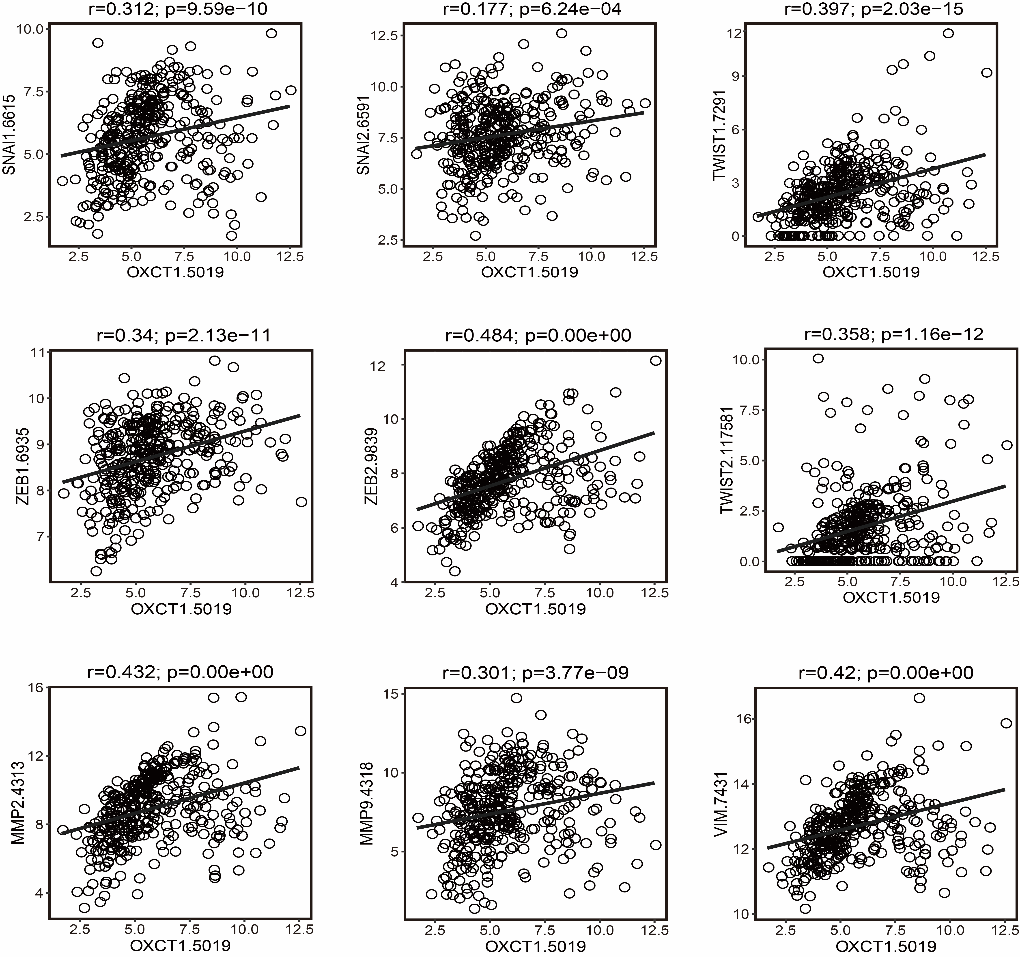


**Supplementary Figure 4. Correlation analysis between OXCT1 and EMT marker expression in HCC.** Analysis of the TCGA LIHC cohort reveals that OXCT1 mRNA expression is significantly positively correlated with the expression of key epithelial-mesenchymal transition (EMT) markers, including SNAI1, SNAI2, TWIST1, TWIST2, ZEB1, ZEB2, MMP2, MMP9, and VIM (all p < 0.001).

**Supplementary Figure 5**


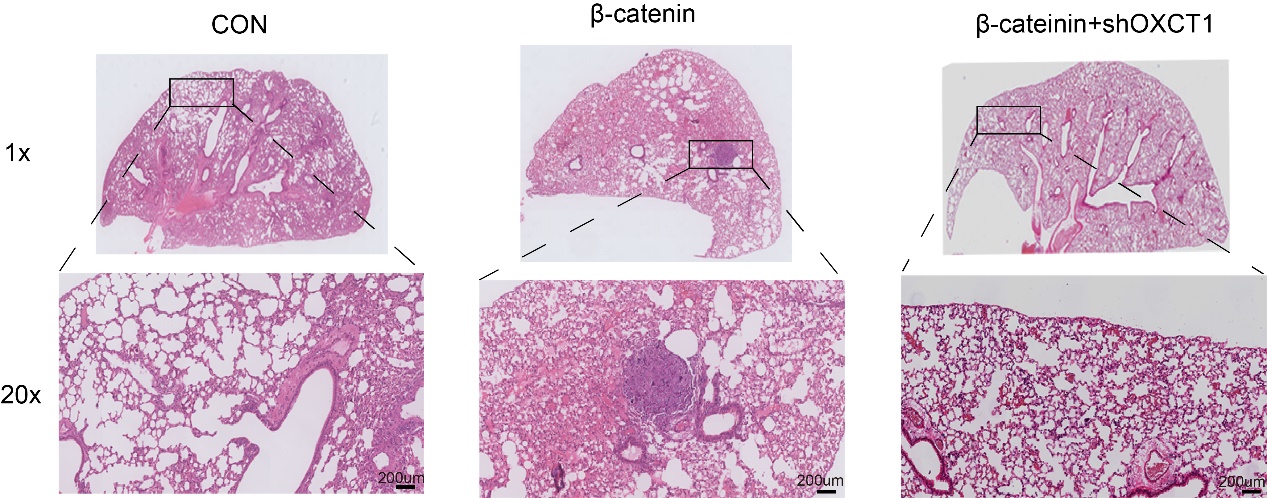


**Supplementary Figure 5. Representative H&E-stained images of lung metastases from the tail vein injection model for the CON, β-catenin, and β-catenin+shOXCT1 groups.**
