# Supplementary material for: β-catenin mutation reprograms ketone body metabolism to drive hepatocellular carcinoma metastasis and resistance to ketogenic therapy via transcriptional activation of OXCT1
Source: Cell Death Dis. 2026 Mar 9;17(1):301. doi: 10.1038/s41419-026-08457-y (PMC13039260; doi:10.1038/s41419-026-08457-y)
Supplement: Supplementary file 2 — Supplementary Table 1: Analysis of the Correlation between OXCT1 Expression and Clinicopathological Data [file 41419_2026_8457_MOESM2_ESM.docx]

Supplementary Table 1: Analysis of the Correlation between OXCT1 Expression and Clinicopathological Data

| OXCT1 expression | | | | | |
| --- | --- | --- | --- | --- | --- |
| Characteristics | Low (185) | High (186) | | | p-value |
| Age (year) |  |  | | | 0.015* |
| Mean | 61.14050 | 57.74054 | | |  |
| SD | 11.95470 | 14.75332 | | |  |
| Gender |  |  | | | 0.605 |
| Male | 127(68.6%) | 123(66.1%) | | |  |
| Female | 58(31.4%) | 63(33.9%) | | |  |
| patient weight |  |  | | | 0.983 |
| Mean | 72.88888889 | 72.84393064 | | |  |
| SD | 18.27484 | 20.58346 | | |  |
| tumor weight |  |  | | | 0.013* |
| Mean | 250.0216216 | 362.8924731 | | |  |
| SD | 338.4816 | 510.82912 | | |  |
| Histologic grade |  |  | | | 0.661 |
| G1 | 31(16.9%) | 24(13.1%) | | |  |
| G2 | 85(46.4%) | 92(50.3%) | | |  |
| G3/4 | 67(36.6%) | 67(36.6%) | | |  |
| Pathological stage |  |  | | | 0.024* |
| Stage I | 96(55.2%) | 75(43.3%) | | |  |
| Stage II | 40(23.0%) | 46(26.6%) | | |  |
| Stage III/IV | 38(21.8%) | 52(30.1%) | | |  |
| T stage |  |  | | | 0.017* |
| T1 | 102(55.1%) | 79(43.2%) | | |  |
| T2 | 44(23.8%) | | 50(27.3%) |  | |
| T3/4 | 39(21.1%) | | 54(29.5%) |  | |
| OS time |  | |  |  | |
| Mean | 879.827027 | | 724.2216216 | 0.039* | |
| SD | 753.88528 | | 692.76385 |  | |

*statistically significant.
