# Supplementary material for: β-catenin mutation reprograms ketone body metabolism to drive hepatocellular carcinoma metastasis and resistance to ketogenic therapy via transcriptional activation of OXCT1
Source: Cell Death Dis. 2026 Mar 9;17(1):301. doi: 10.1038/s41419-026-08457-y (PMC13039260; doi:10.1038/s41419-026-08457-y)
Supplement: Supplementary file 3 — Original Western blots [file 41419_2026_8457_MOESM3_ESM.docx]

**Figure 2 A**


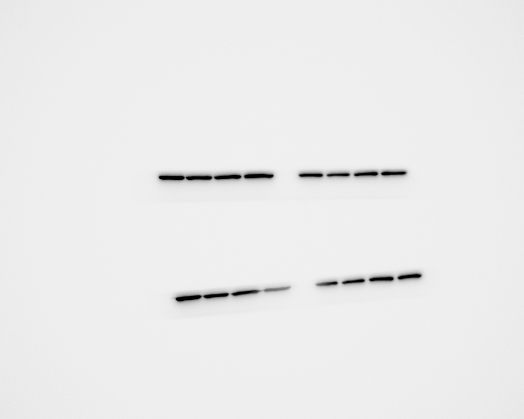
 β-actin


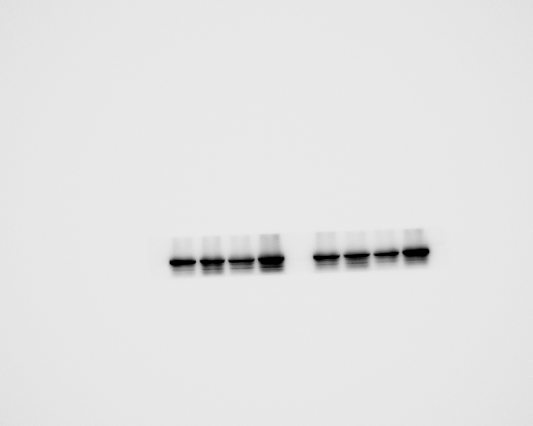
β-catenin


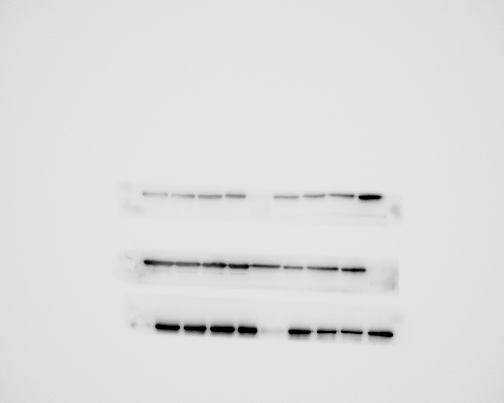
BDH1


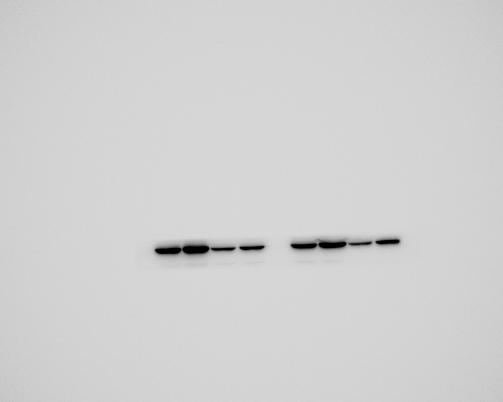
Hmgcs2


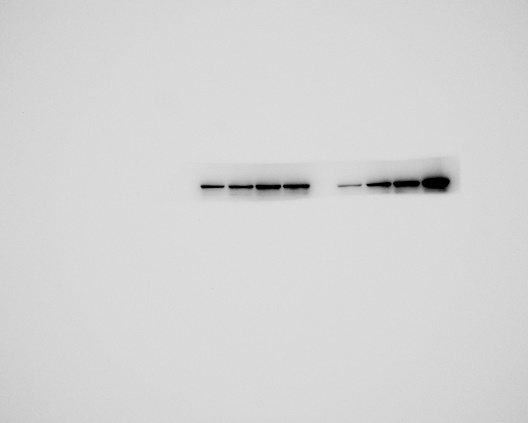
ppara

**Figure 3 B**


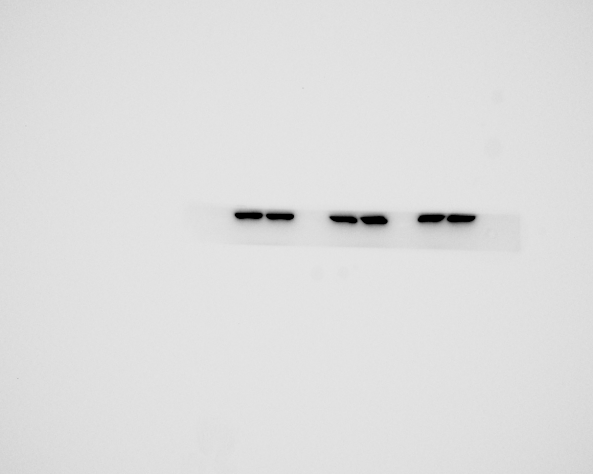
β-actin


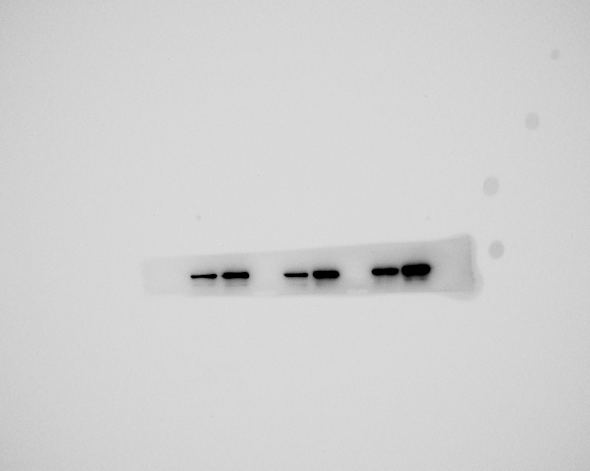
β-catenin


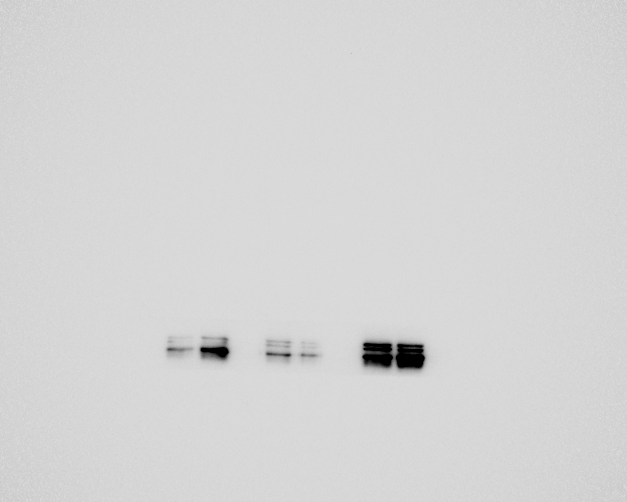
3B-OXCT1

**Figure 3 D**


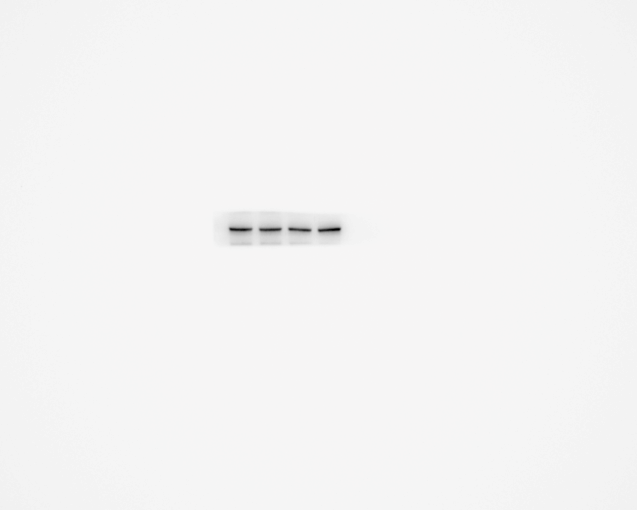
β-actin


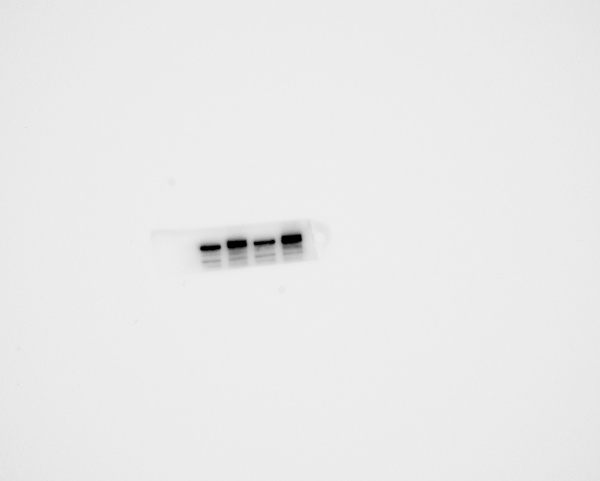
β-catenin


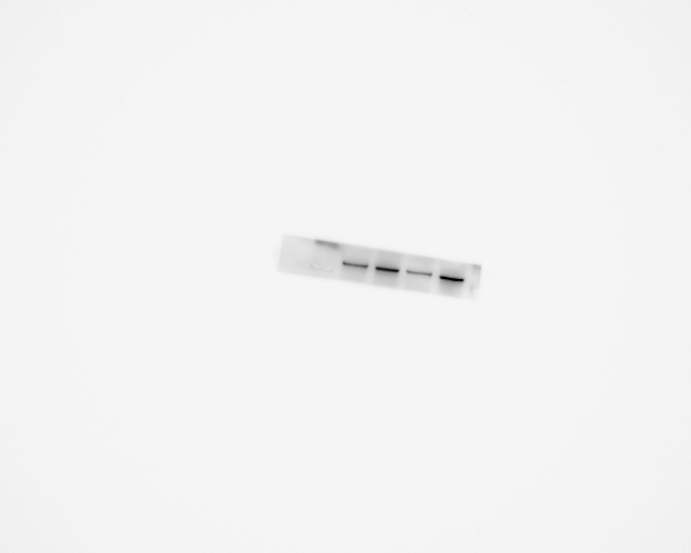
OXCT1

**Figure 3 F**


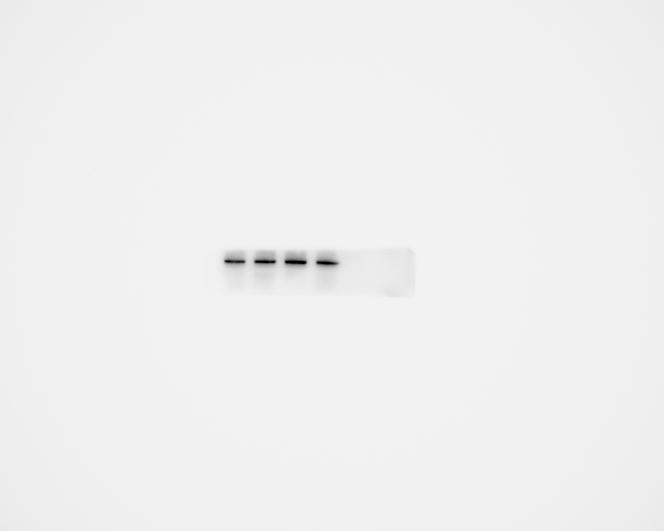
β-actin


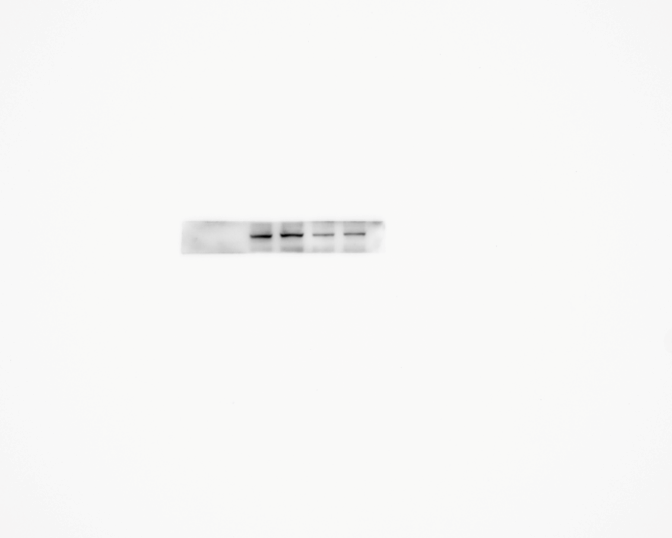
β-catenin


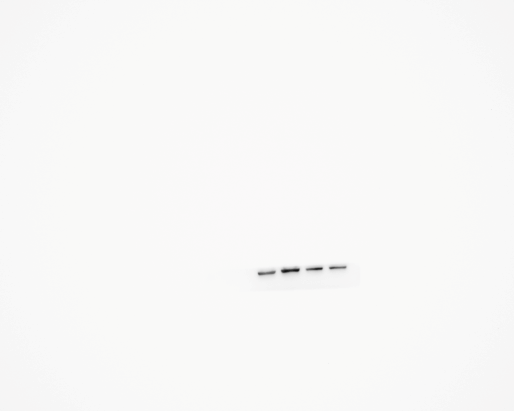
OXCT1

**Figure 3 H**


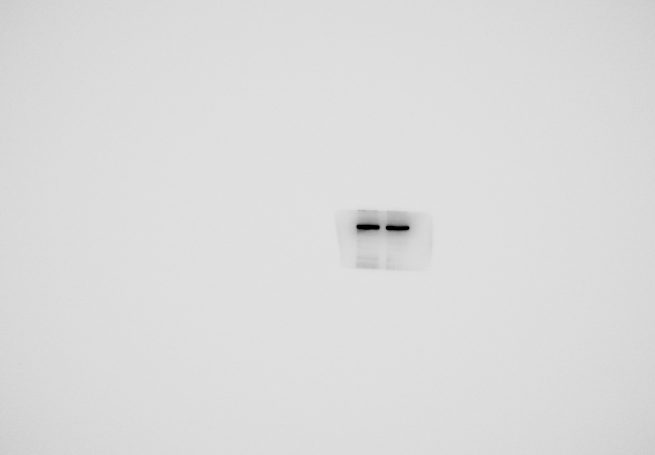
β-actin


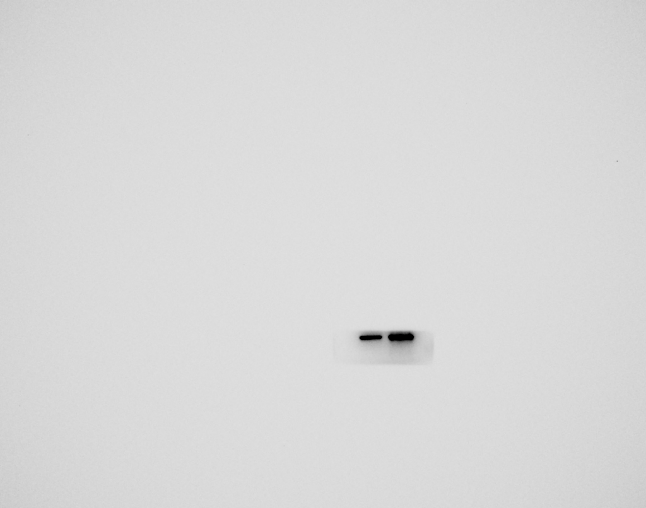
LEF1


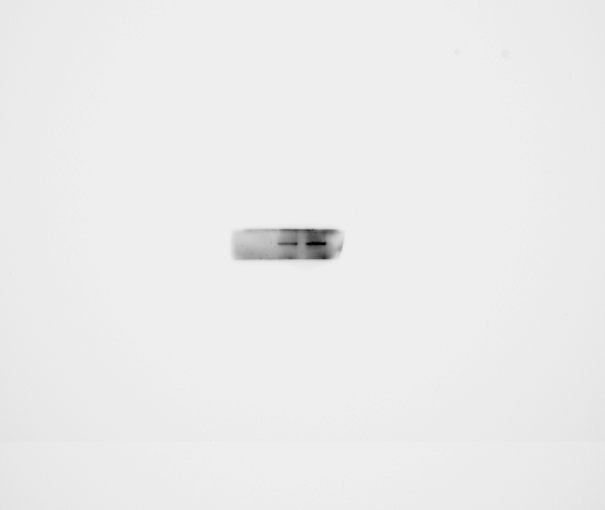
OXCT1

**Figure 3 I**


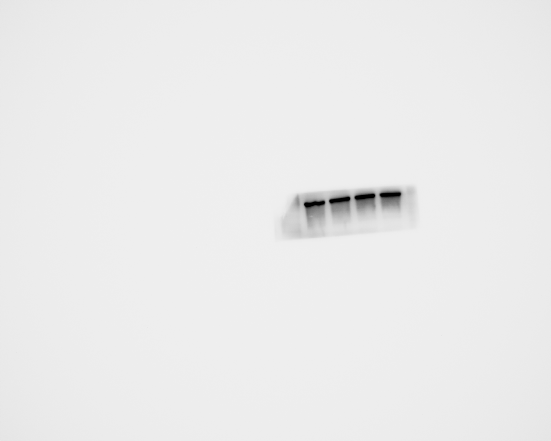
β-actin


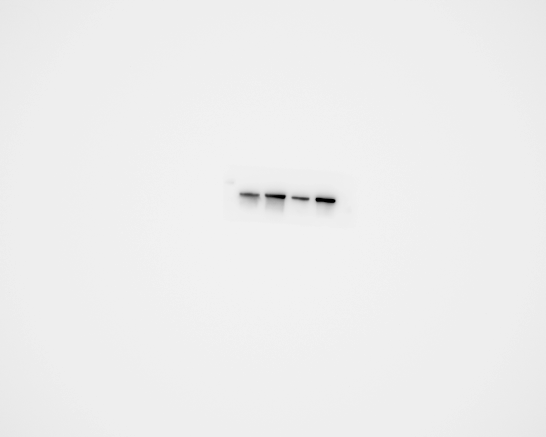
β-catenin

**
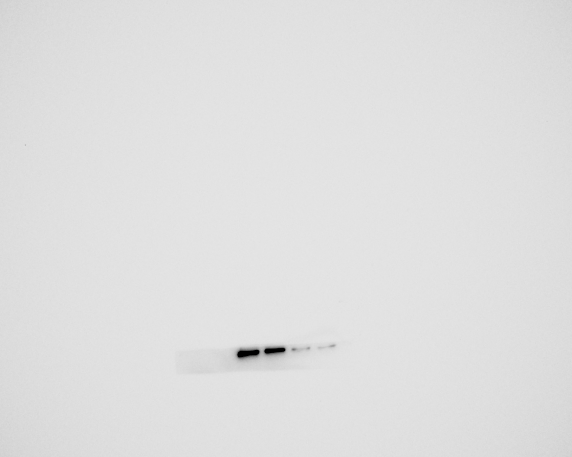
LEF1**


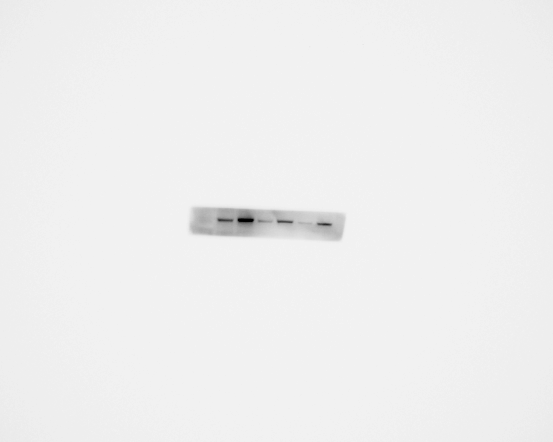
OXCT1

**Figure 3 J**


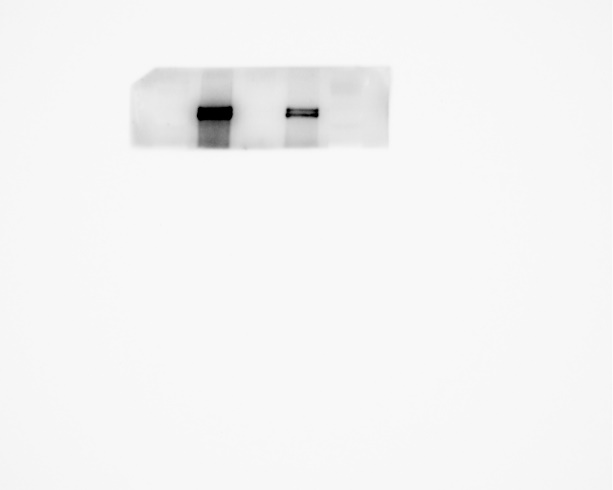
β-catenin


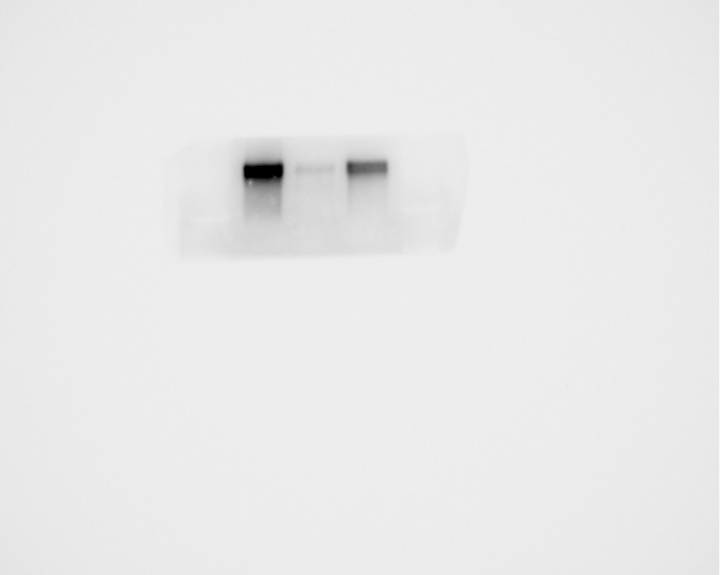
LEF1

**Figure 4 A**

**
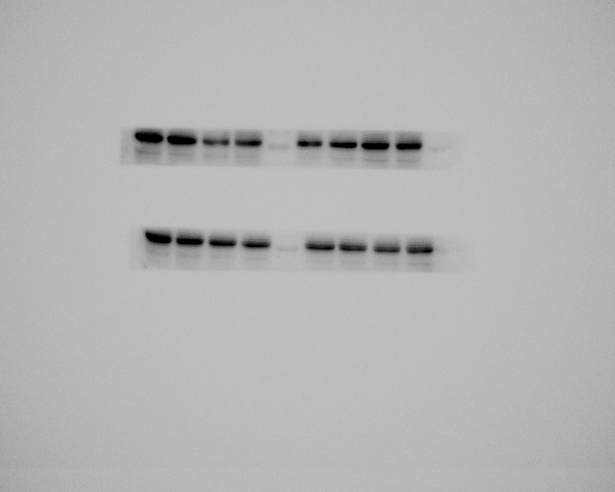
**β-actin

**
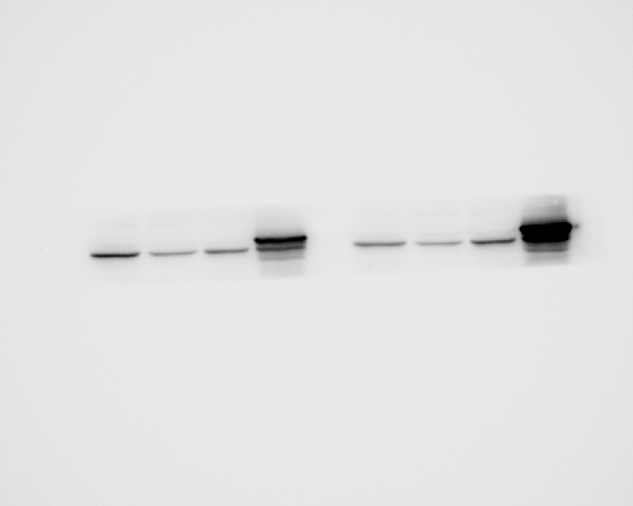
**OXCT1

**Figure 4 C**


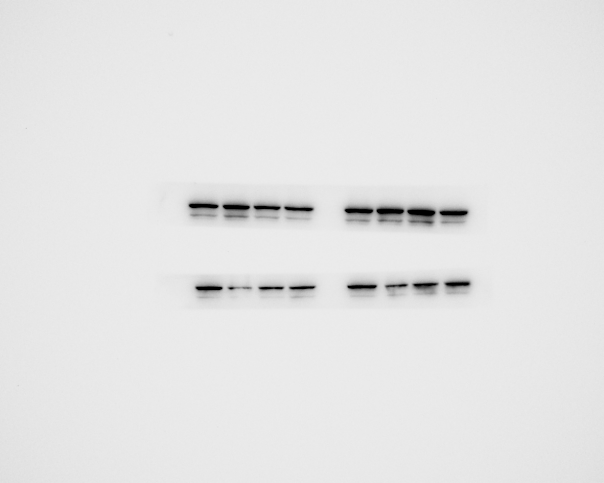
β-actin


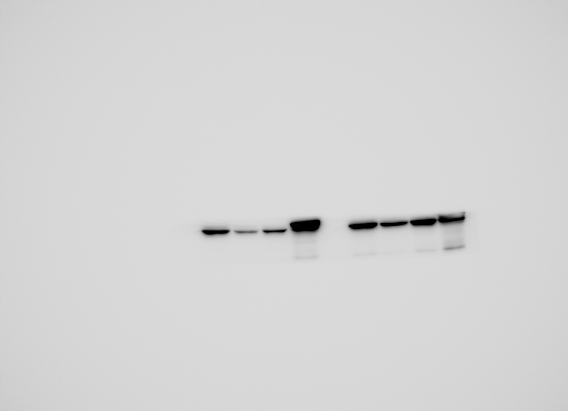
OXCT1

**Figure 5 C**


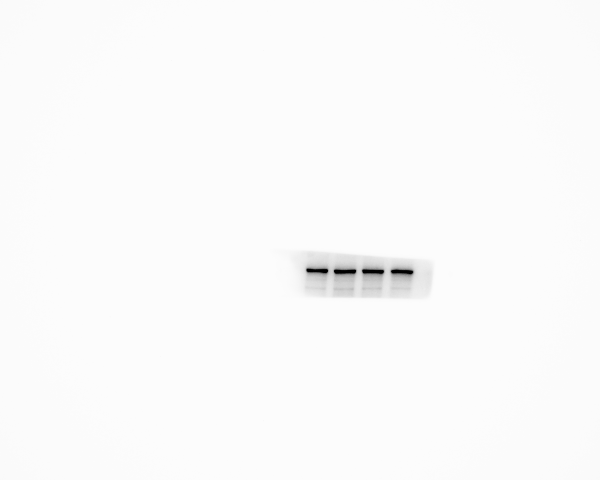
β-actin


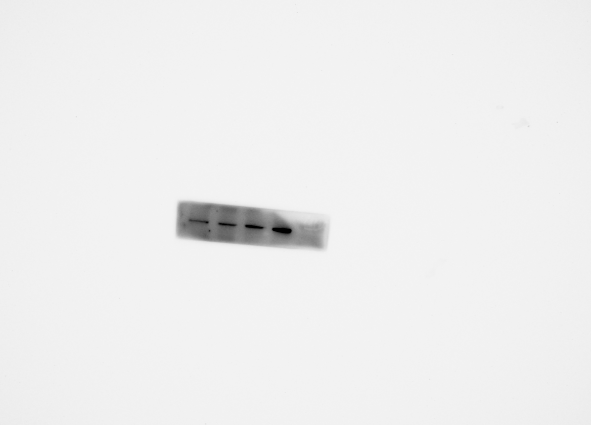
mmp2


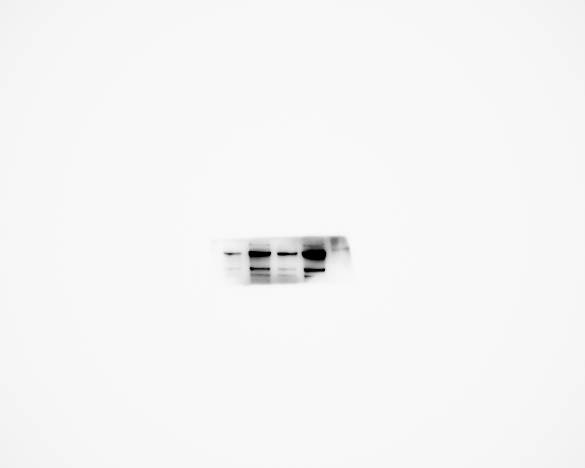
OXCT1


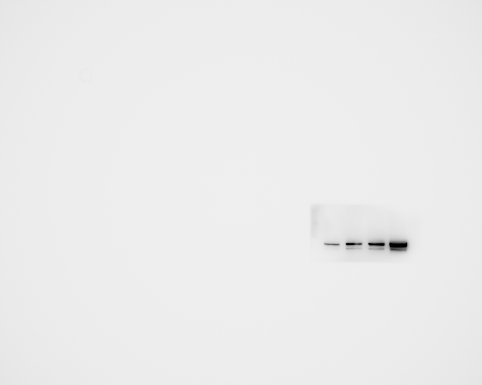
P-STAT3


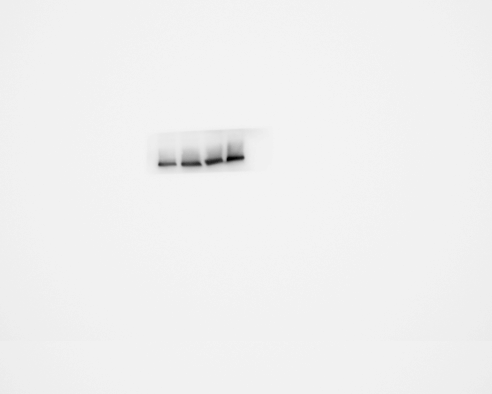
Snail


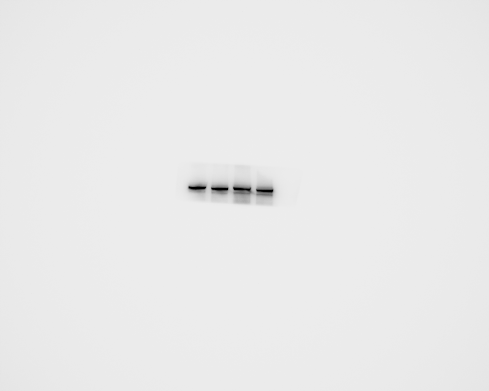
stat3


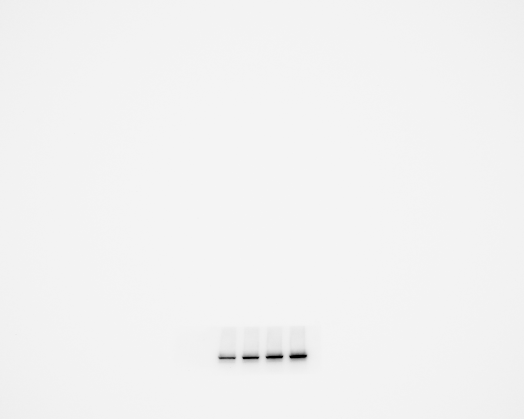
vimentin


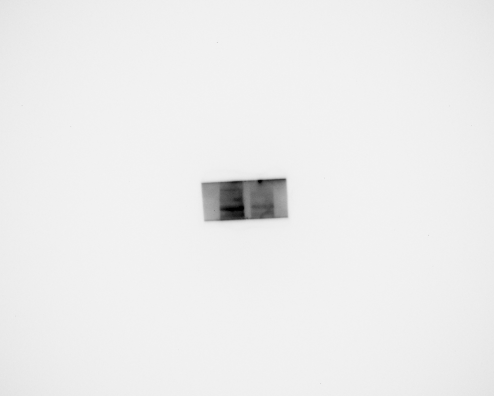
E-cadherin

**Figure 5 D**


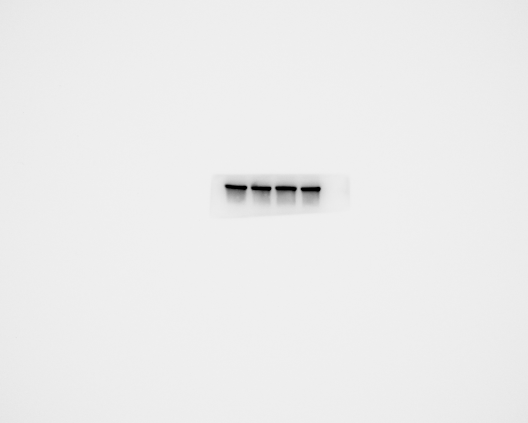
β-actin


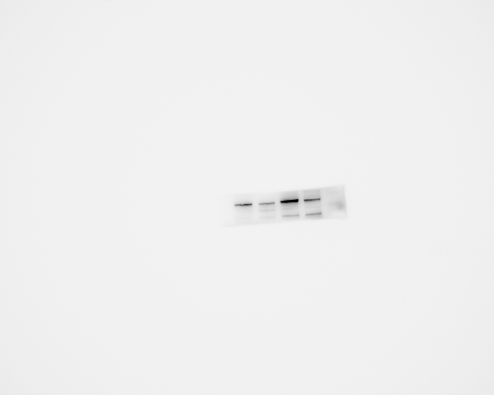
OXCT1


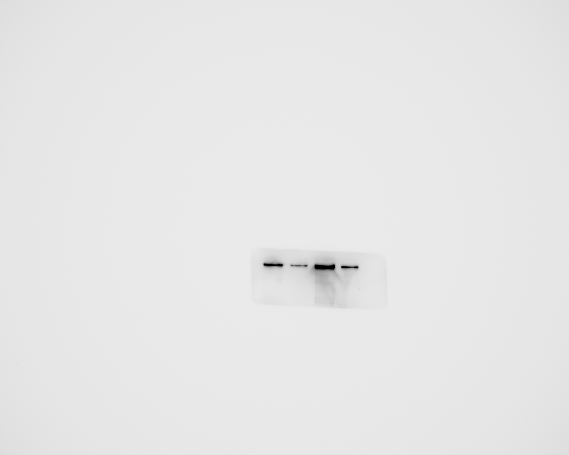
MMP2


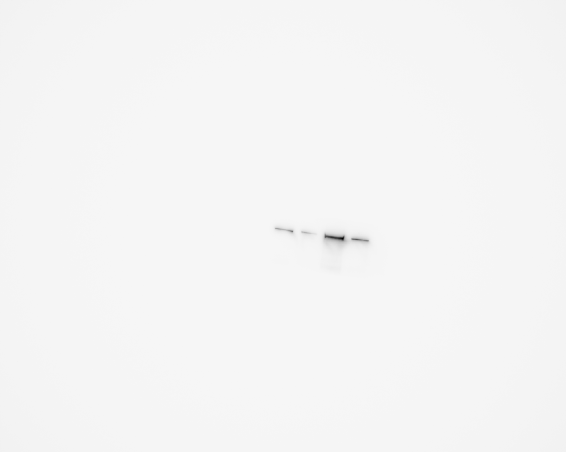
snai1


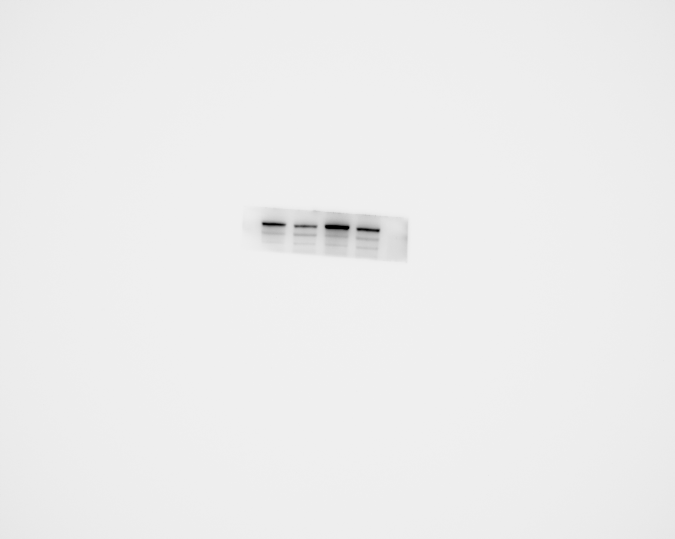
vimentin


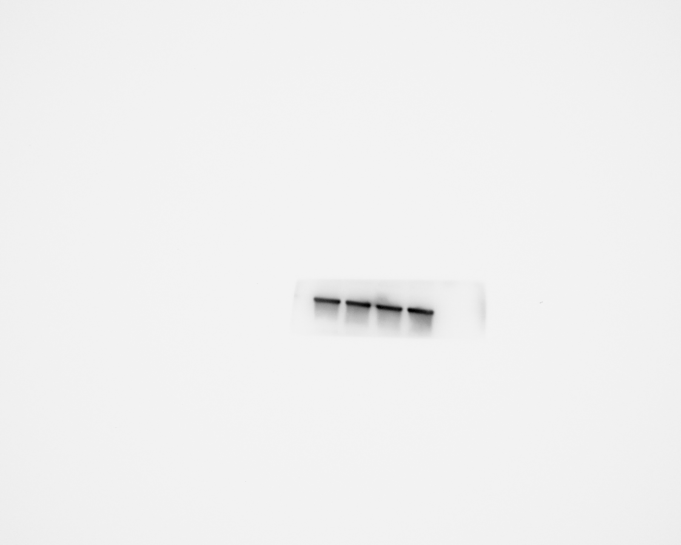
stat3


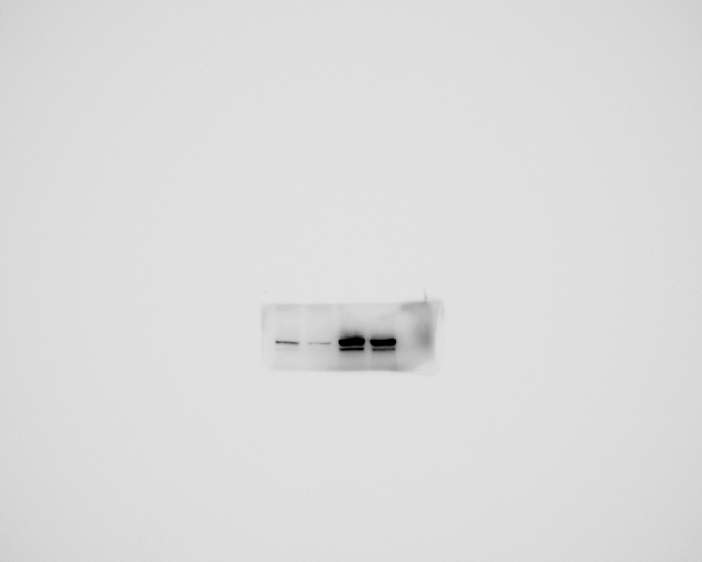
p-stat3


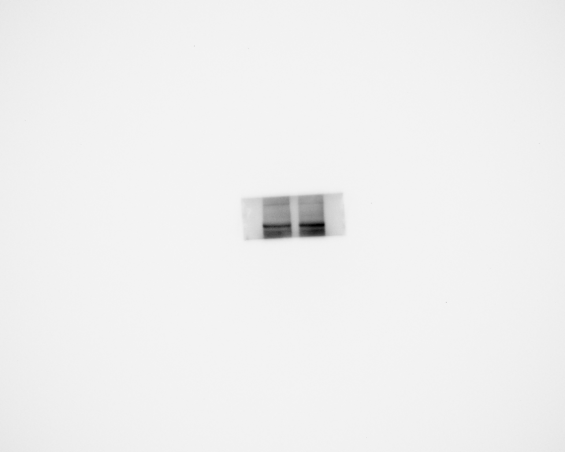
E-cadherin

**Figure 5 E**


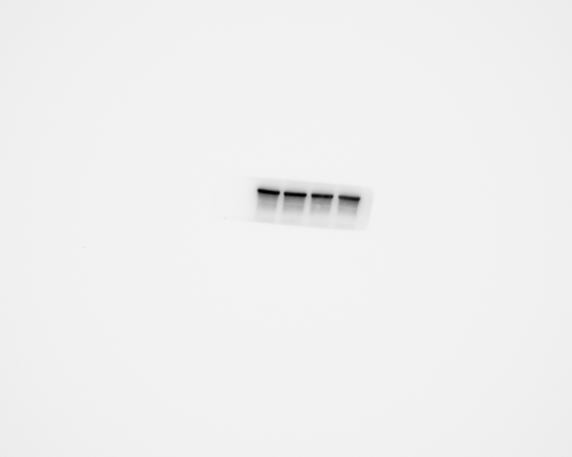
β-actin


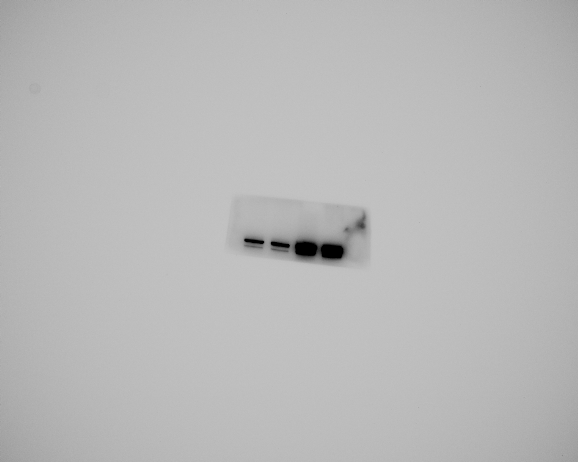
oxct1


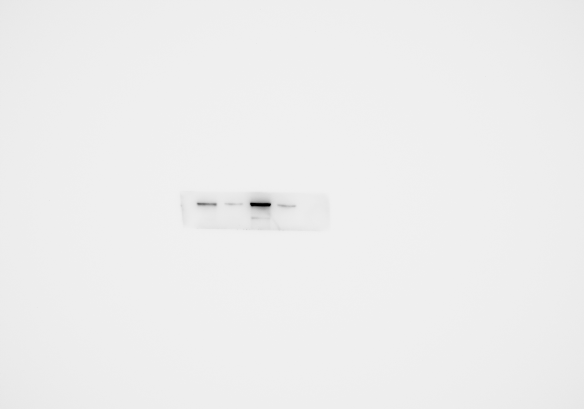
Snai1


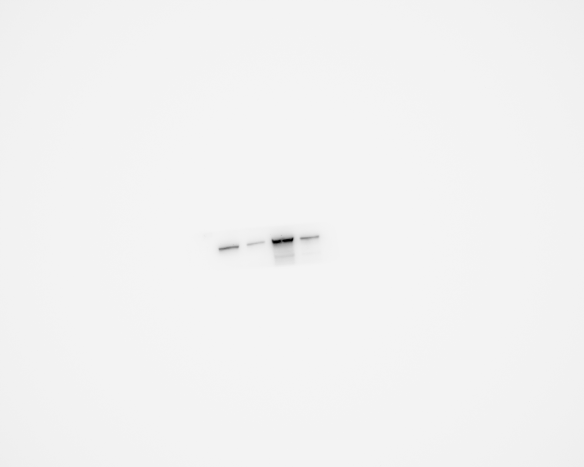
mmp2


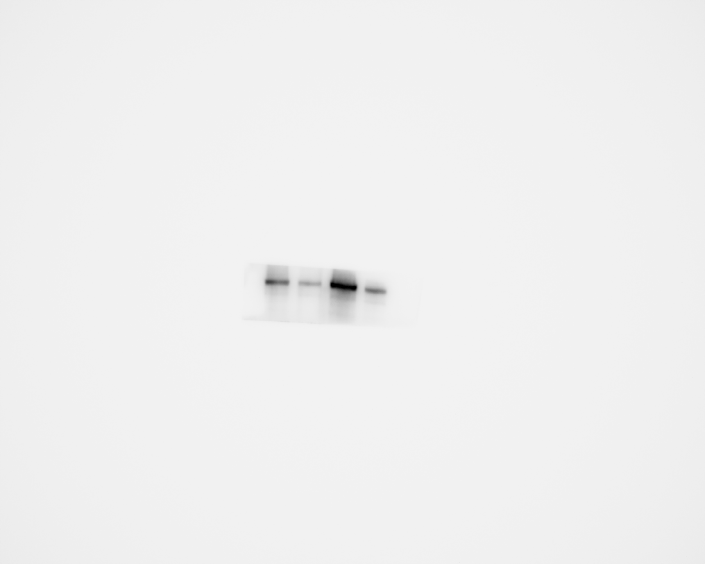
Vimentin


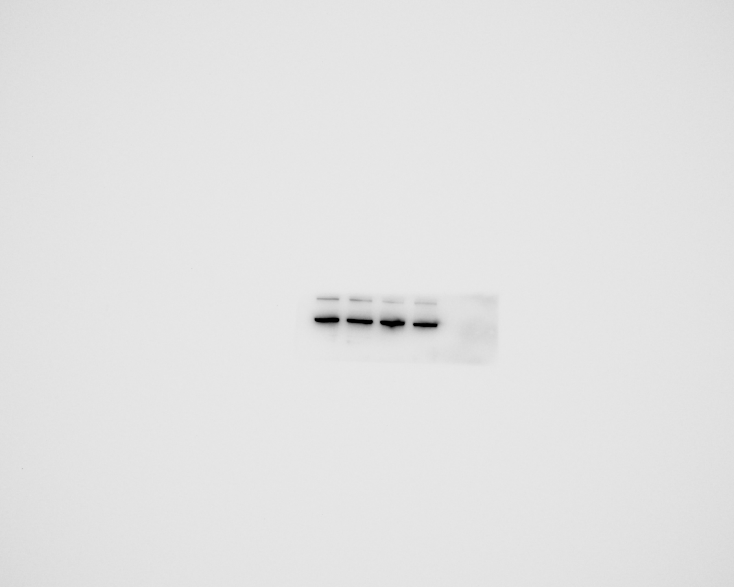
stat3


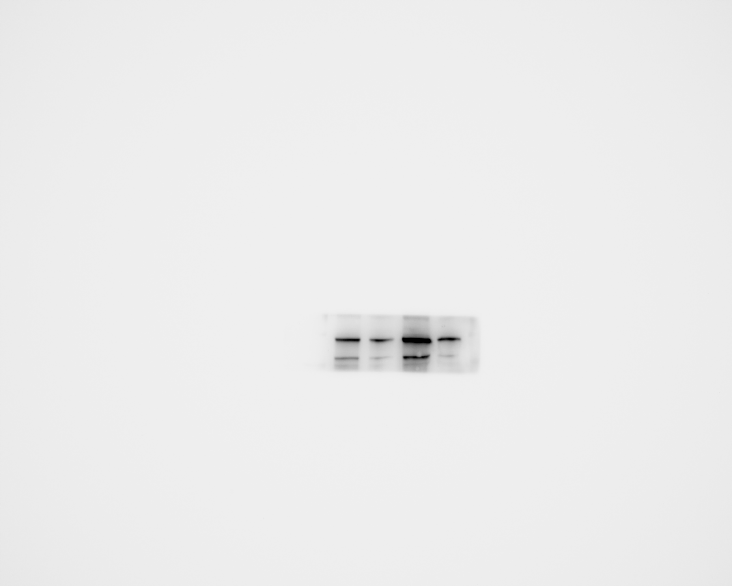
p-stat3


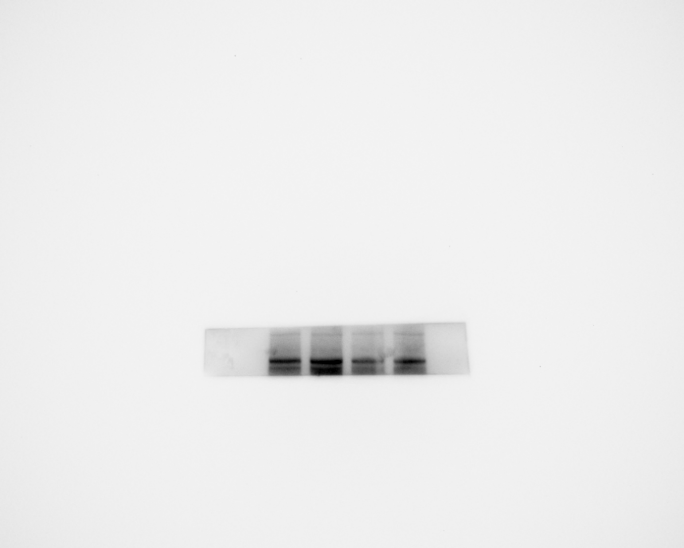
E-cadherin

**Figure 6 A**


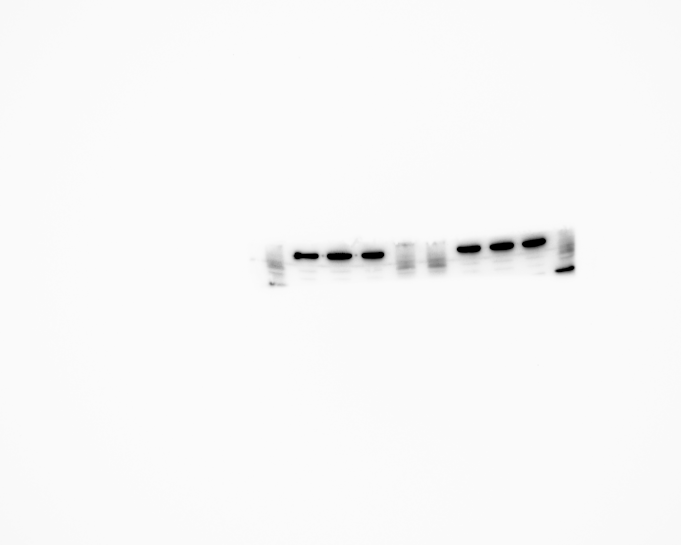
β-actin


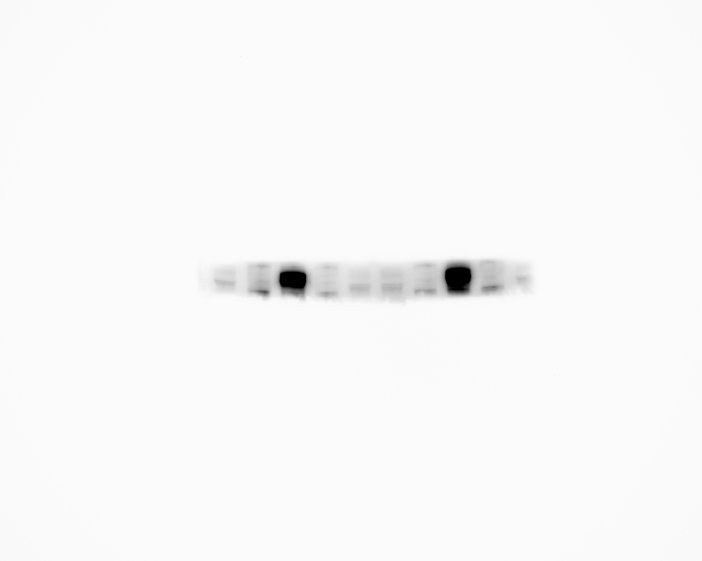
oxct1


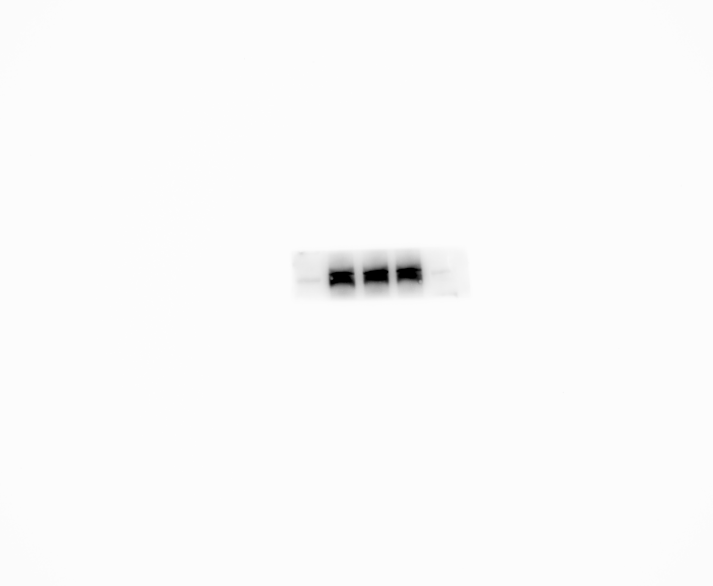
β-catenin

**Figure 6 D**


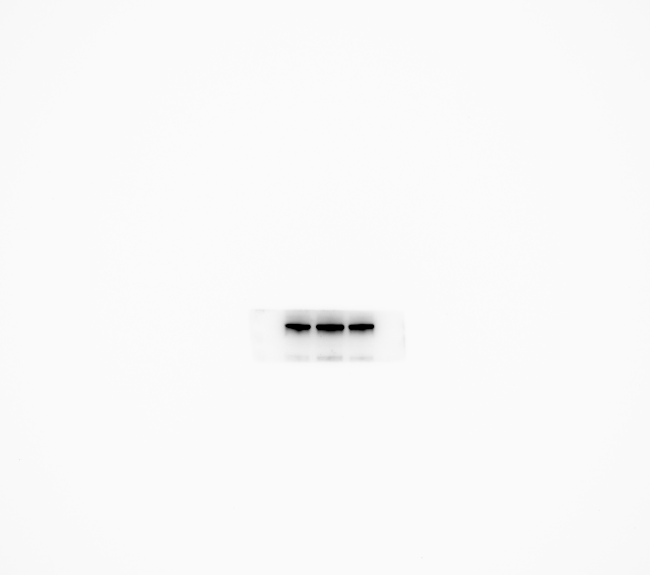
β-actin


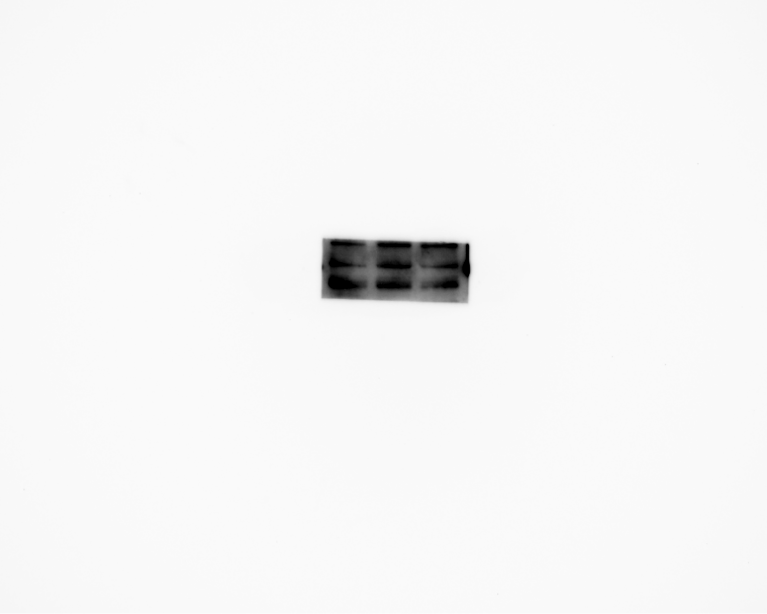
Vimentin


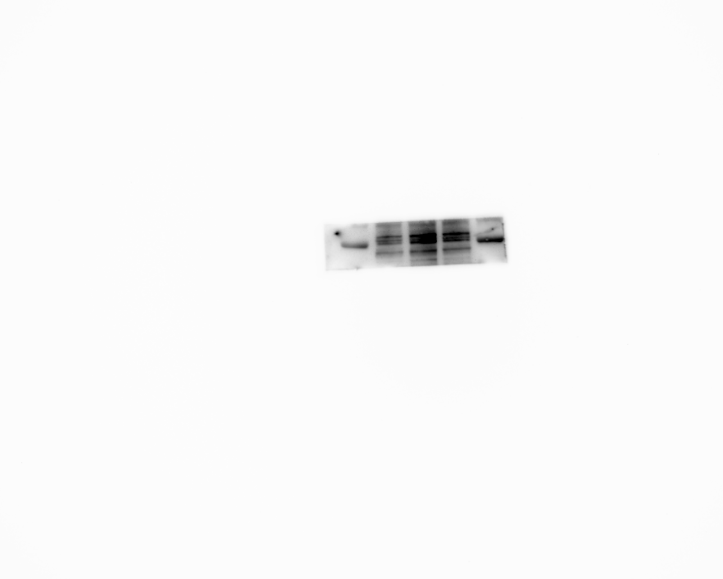
mmp2


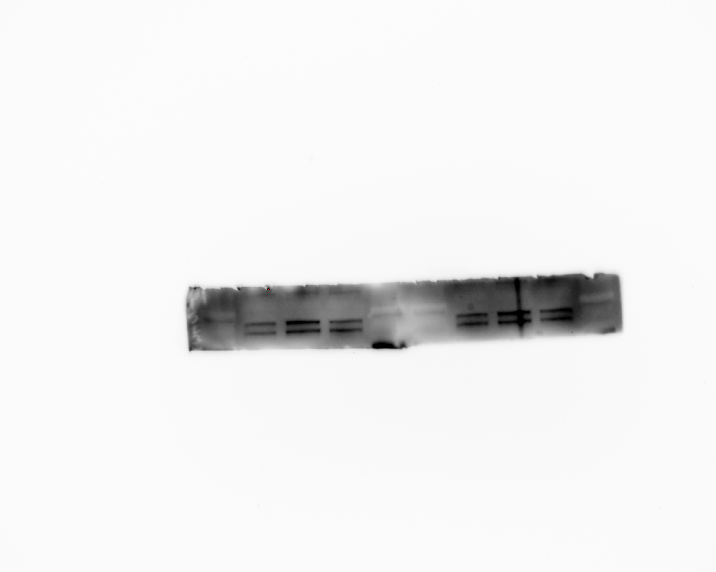
p-stat3


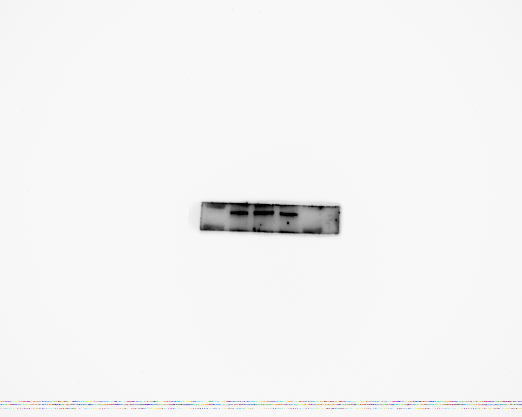
stat3


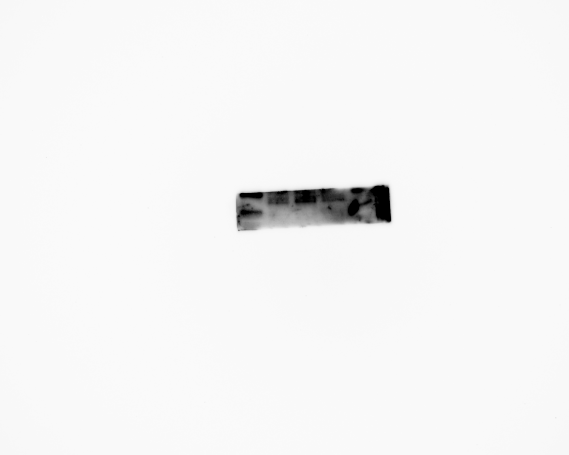
Snail


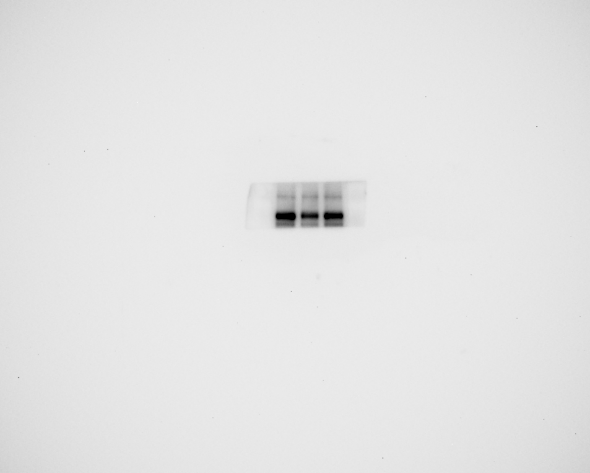
E-cadherin

**Figure 6 F**

**
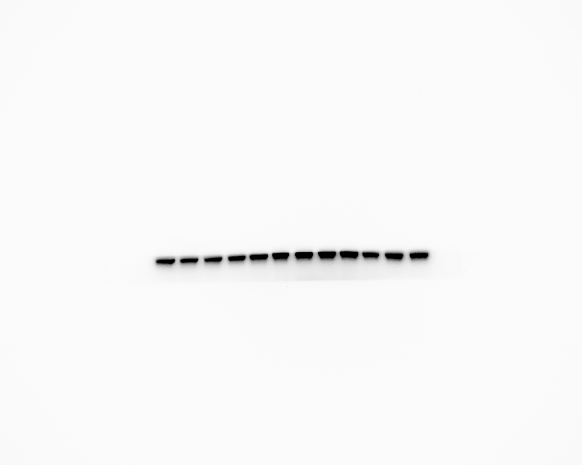
**β-actin


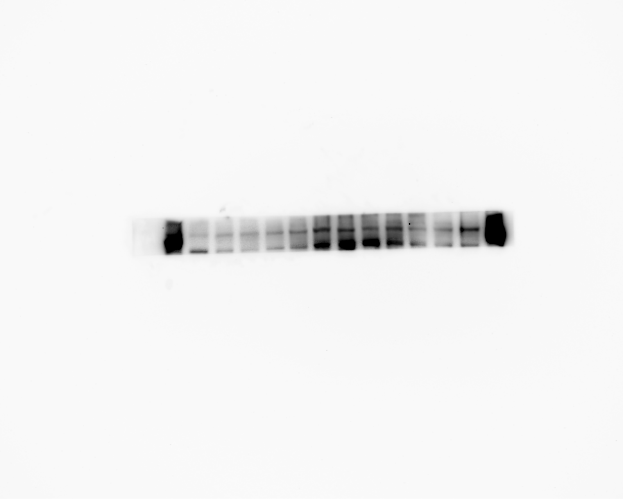
Vimentin


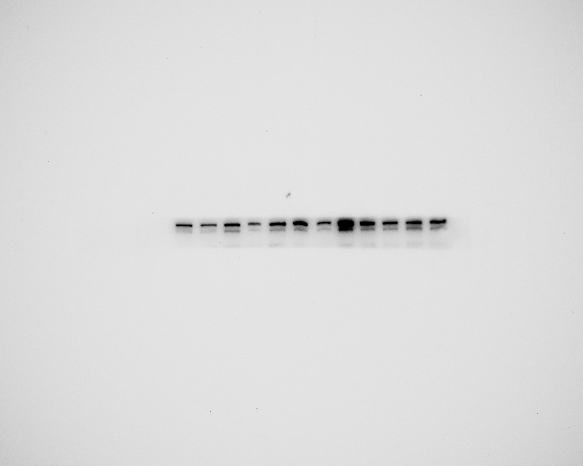
pstat3


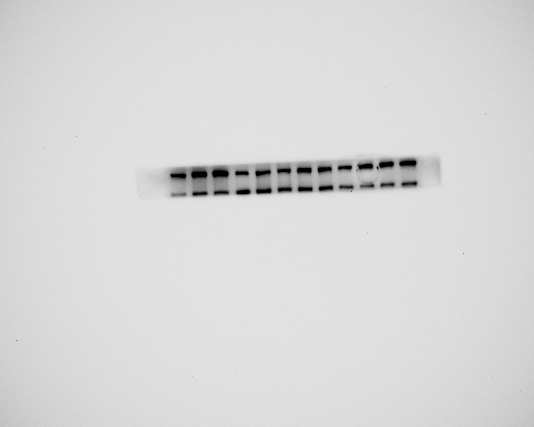
stat3


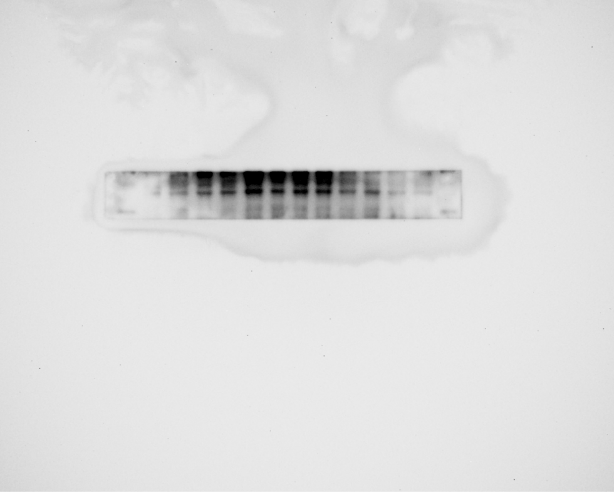
snai1


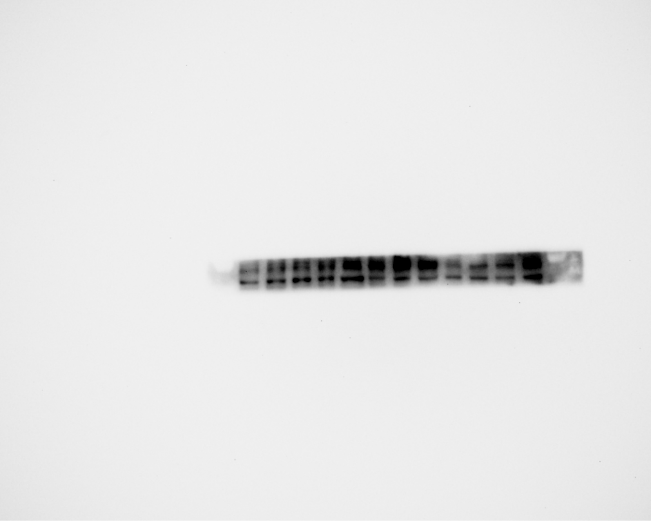
mmp2


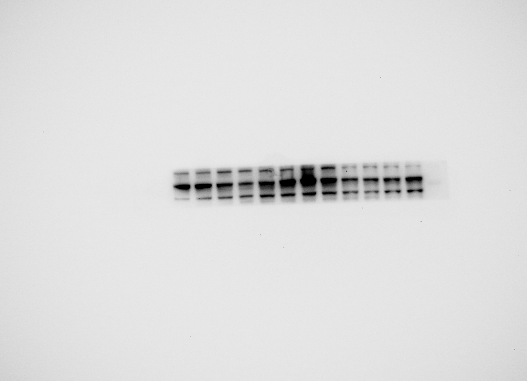
OXCT1


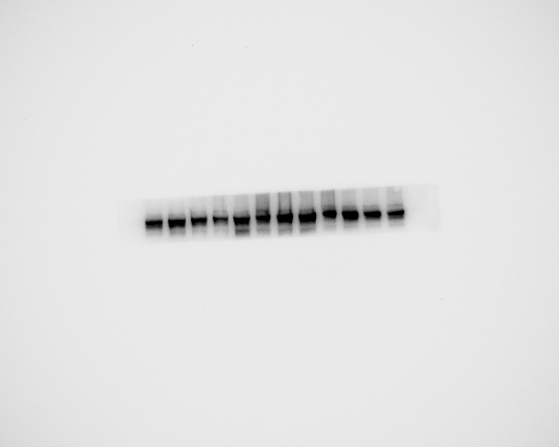
β-catenin


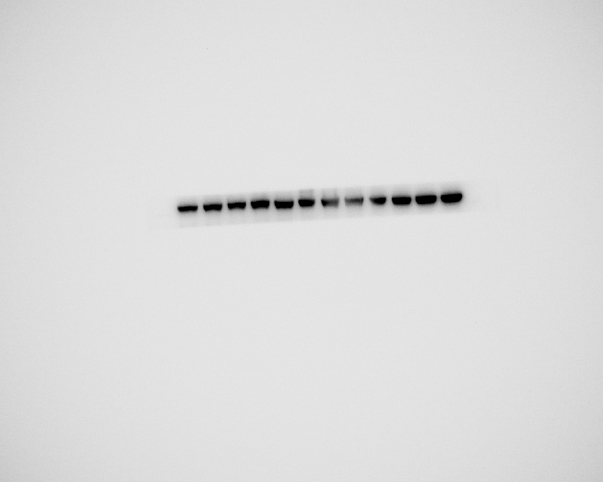
β-actin


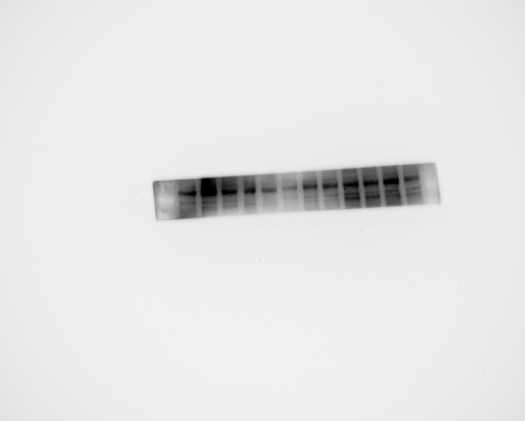
E-cadherin

**Figure 7 G**

**
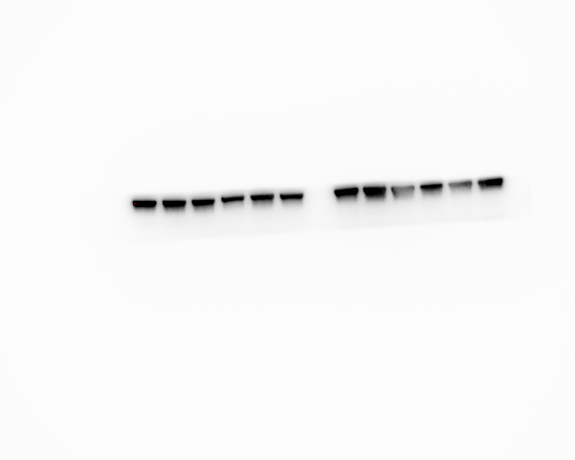
**β-actin

**
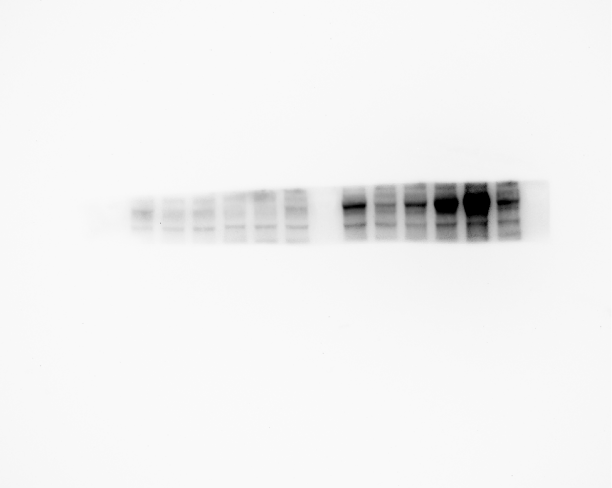
OXCT1**

**
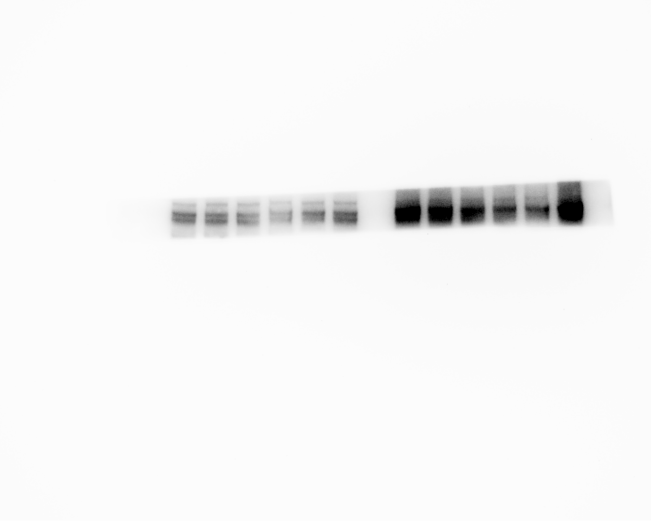
**β-catenin

**Figure 7 I**

**
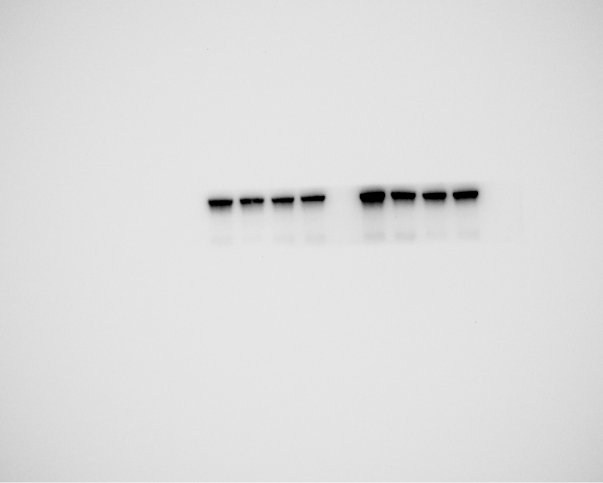
**ND-β-actin


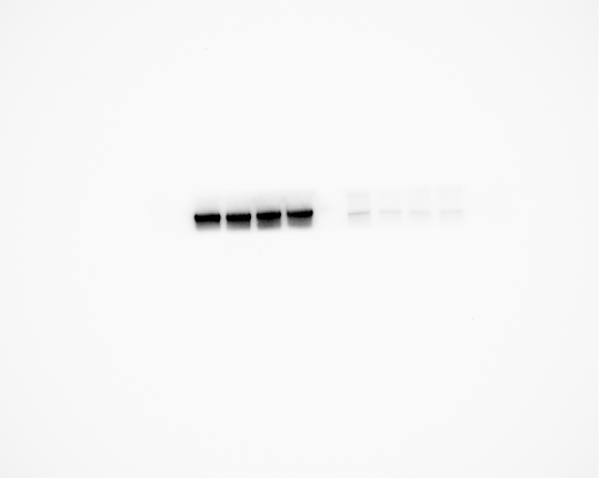
ND-OXCT1


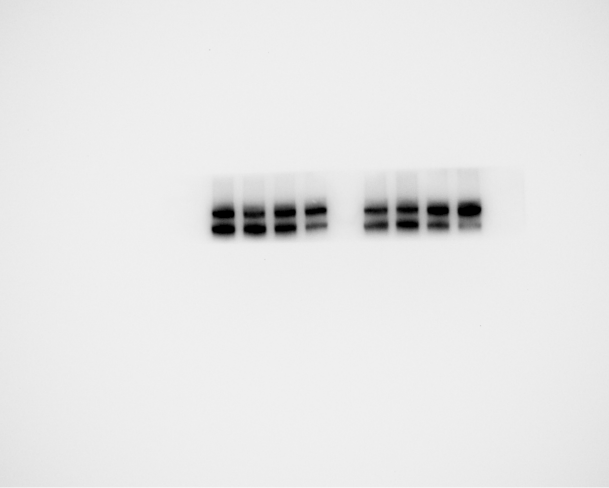
ND-β-catenin


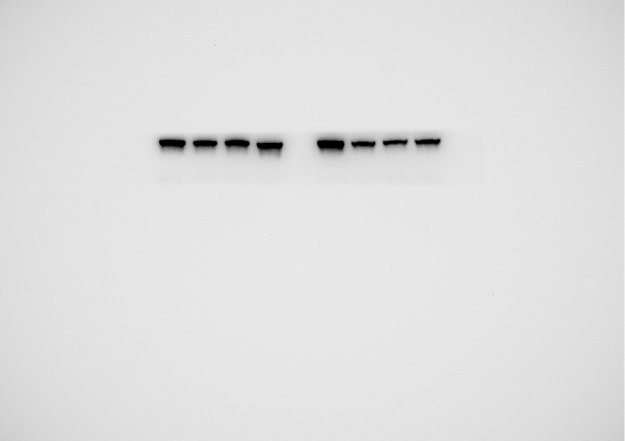
KD-β-actin


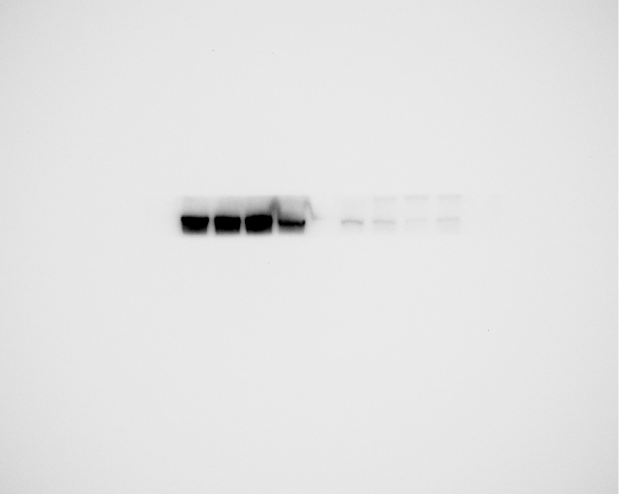
KD-OXCT1


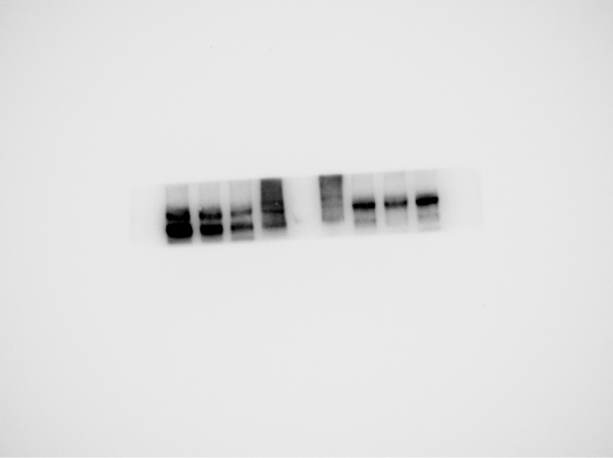
KD-β-catenin

**Supplementary Figure 1B**


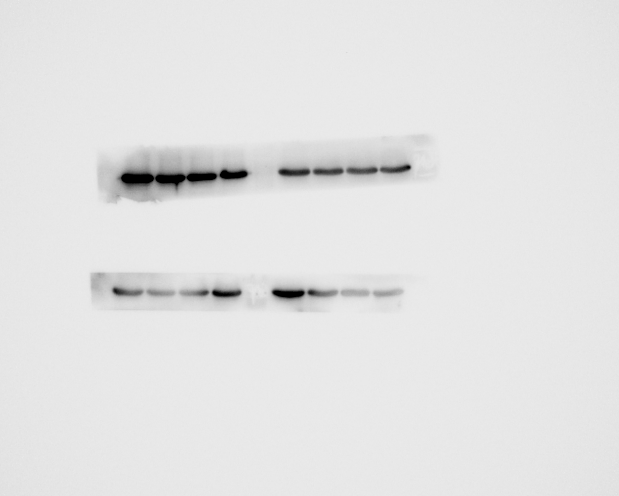
β-actin


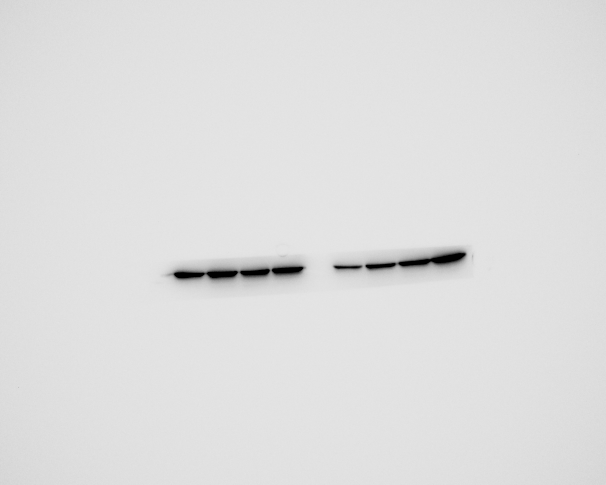
OXCT1


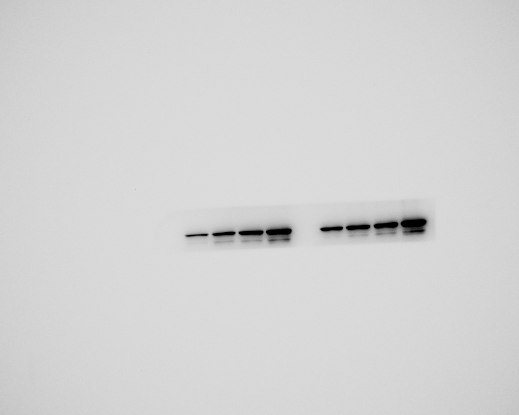
β-catenin

**Supplementary Figure 1C**


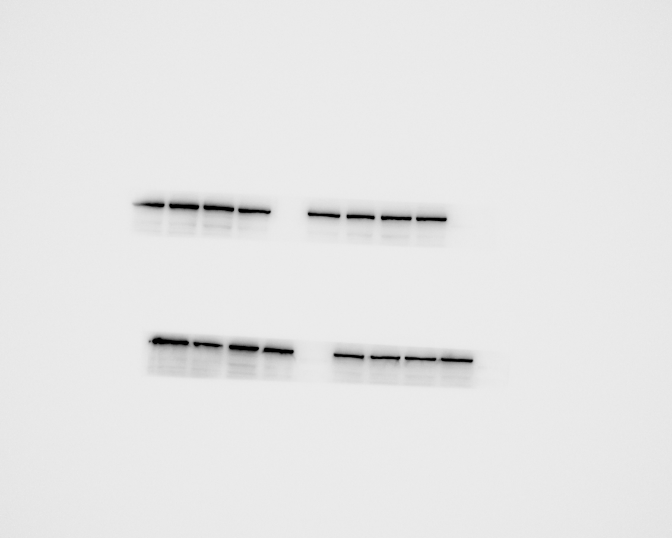
β-actin


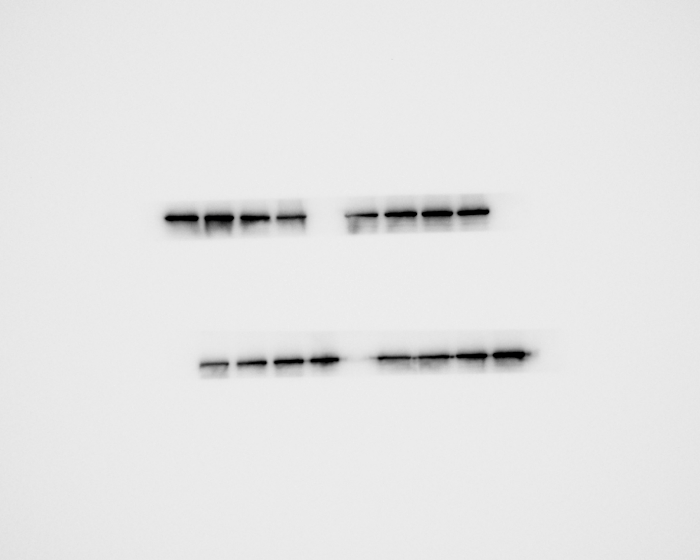
OXCT1


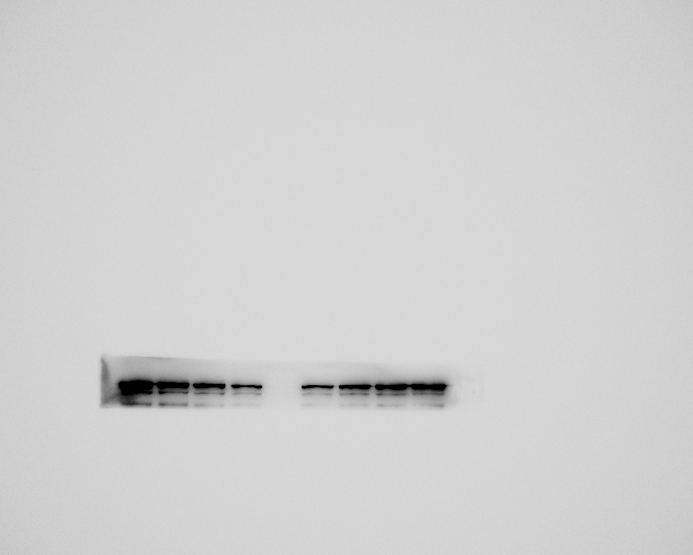
β-catenin

**Supplementary Figure 1C**


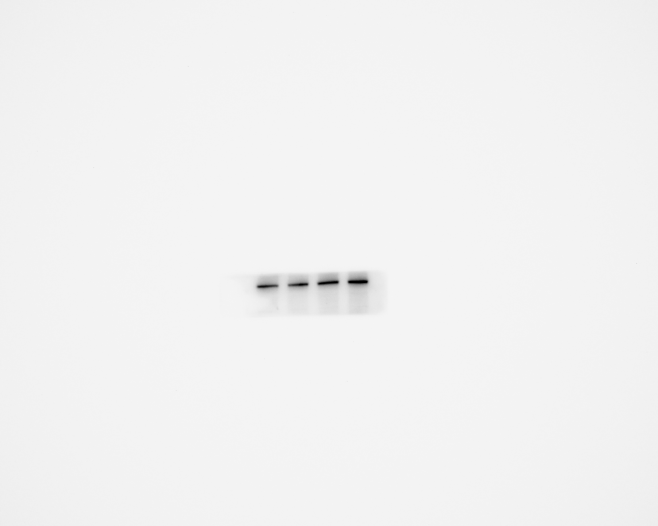
β-actin


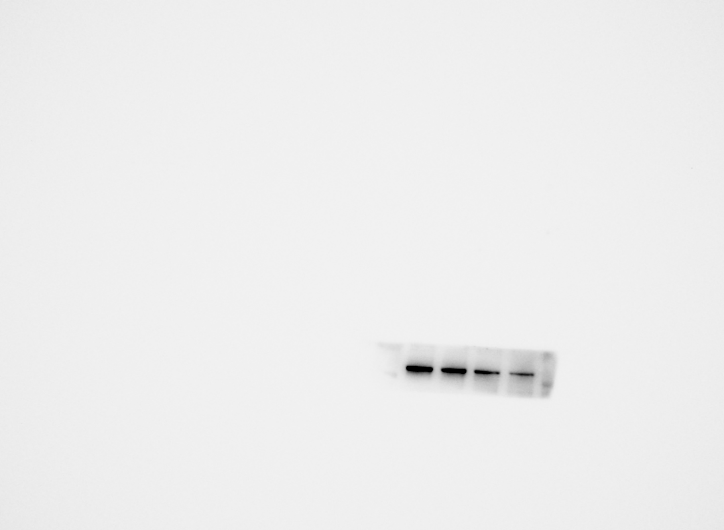
OXCT1


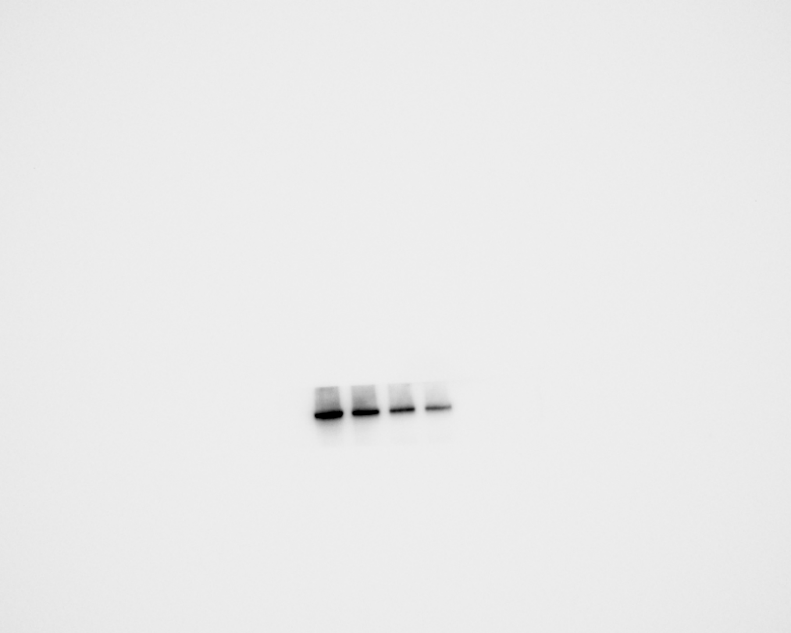
β-catenin

**Supplementary Figure 1E**


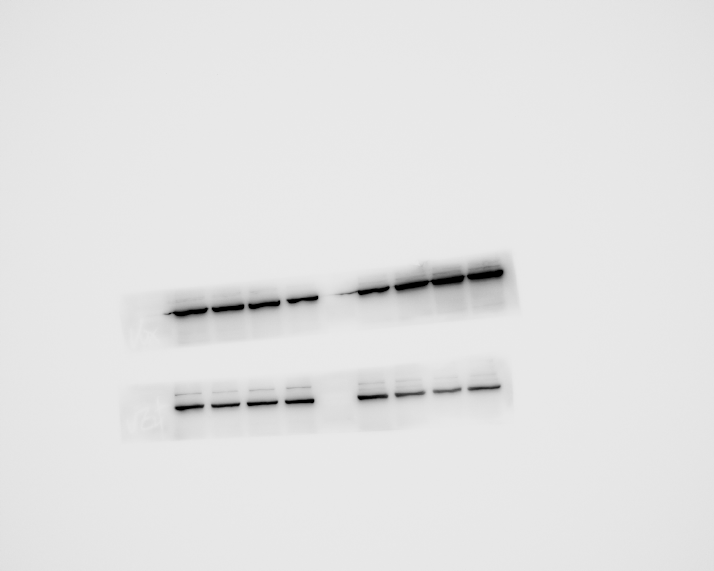
β-actin


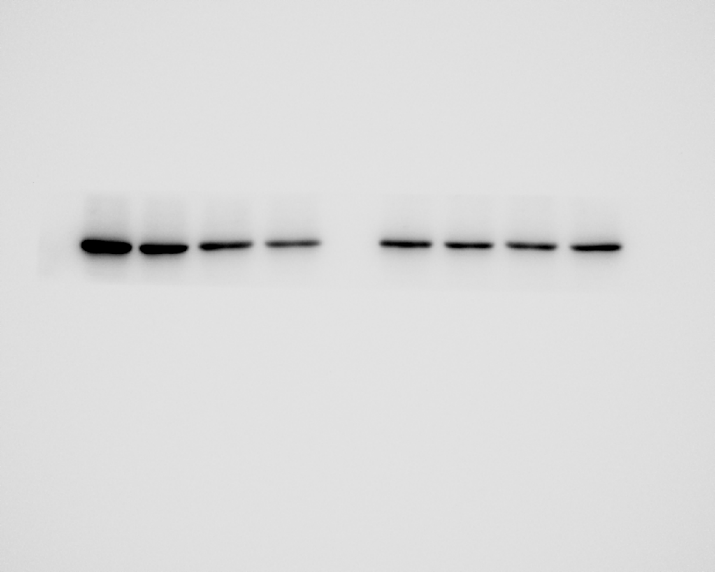
OXCT1


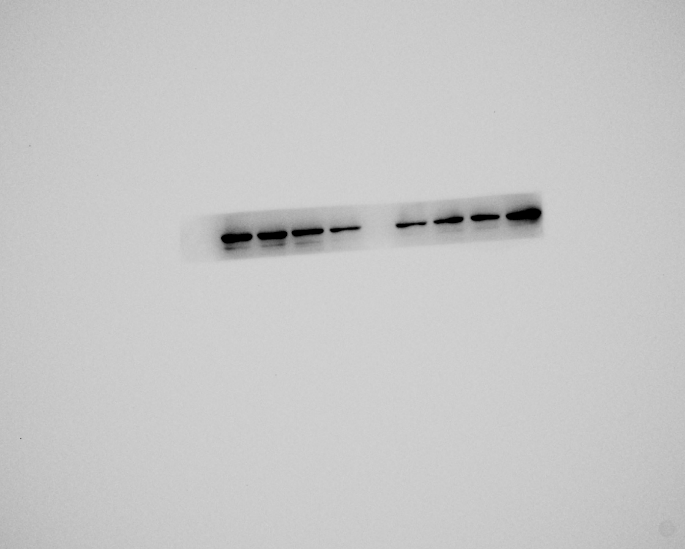
β-catenin

**Supplementary Figure 2D**


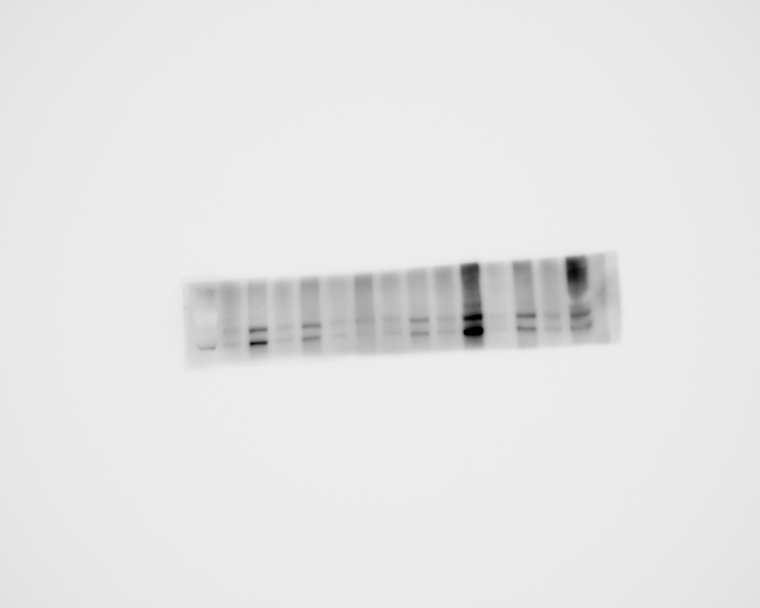
OXCT1


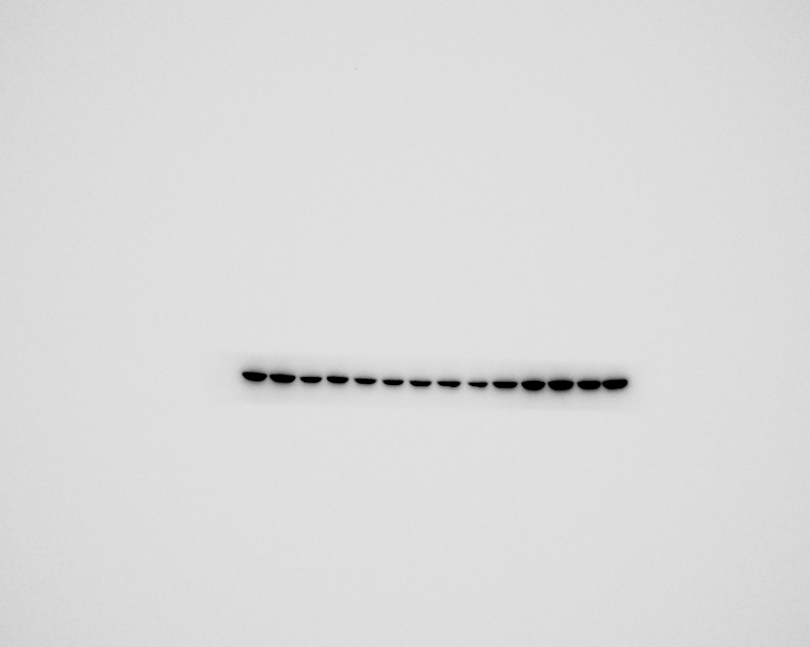
β-actin
